# Transcriptional Regulation by Protein Kinase A in Cryptococcus neoformans

**DOI:** 10.1371/journal.ppat.0030042

**Published:** 2007-03-16

**Authors:** Guanggan Hu, Barbara R Steen, Tianshun Lian, Anita P Sham, Nicola Tam, Kristin L Tangen, James W Kronstad

**Affiliations:** The Michael Smith Laboratories, The University of British Columbia, Vancouver, British Columbia, Canada; Duke University Medical Center, United States of America

## Abstract

A defect in the *PKA1* gene encoding the catalytic subunit of cyclic adenosine 5′-monophosphate (cAMP)–dependent protein kinase A (PKA) is known to reduce capsule size and attenuate virulence in the fungal pathogen Cryptococcus neoformans. Conversely, loss of the PKA regulatory subunit encoded by *pkr1* results in overproduction of capsule and hypervirulence. We compared the transcriptomes between the *pka1* and *pkr1* mutants and a wild-type strain, and found that PKA influences transcript levels for genes involved in cell wall synthesis, transport functions such as iron uptake, the tricarboxylic acid cycle, and glycolysis. Among the myriad of transcriptional changes in the mutants, we also identified differential expression of ribosomal protein genes, genes encoding stress and chaperone functions, and genes for secretory pathway components and phospholipid synthesis. The transcriptional influence of PKA on these functions was reminiscent of the linkage between transcription, endoplasmic reticulum stress, and the unfolded protein response in Saccharomyces cerevisiae. Functional analyses confirmed that the PKA mutants have a differential response to temperature stress, caffeine, and lithium, and that secretion inhibitors block capsule production. Importantly, we also found that lithium treatment limits capsule size, thus reinforcing potential connections between this virulence trait and inositol and phospholipid metabolism. In addition, deletion of a PKA-regulated gene, *OVA1,* revealed an epistatic relationship with *pka1* in the control of capsule size and melanin formation. *OVA1* encodes a putative phosphatidylethanolamine-binding protein that appears to negatively influence capsule production and melanin accumulation. Overall, these findings support a role for PKA in regulating the delivery of virulence factors such as the capsular polysaccharide to the cell surface and serve to highlight the importance of secretion and phospholipid metabolism as potential targets for anti-cryptococcal therapy.

## Author Summary

The ability of pathogens to regulate the export of proteins and other macromolecules is an important aspect of the infection process. The fungal pathogen Cryptococcus neoformans causes life-threatening infections in individuals with AIDS and delivers several virulence factors to the cell surface. These factors include polysaccharide material that forms a prominent capsule as well as the enzyme laccase that produces a protective layer of melanin in the cell wall. The cyclic adenosine 5′-monophosphate (cAMP) signaling pathway in C. neoformans plays a key role in sensing conditions such as nutrient availability to control expression of virulence factors, and defects in the pathway lead to attenuated or accentuated disease. Transcriptional profiling identified a regulatory link between the cAMP pathway and components of the machinery for transport to the cell surface. Studies with secretion inhibitors and with gene disruption mutants further supported connections between cAMP signaling, export functions, and the delivery of capsule and protein cargo outside the cell. These studies indicate that C. neoformans is a useful model for studying the regulation of secretion because of its particular dependence on this process for infection. In general, this work highlights the fact that components of the secretion machinery represent attractive targets for therapeutic measures to control fungal and other diseases.

## Introduction

Cryptococcus neoformans is a basidiomycete fungal pathogen that infects both immunocompromised and immunocompetent individuals to cause meningioencephalitis [1]. A variety of virulence factors have been characterized, including the formation of a polysaccharide capsule, the production of the pigment melanin in the cell wall, the ability to grow at 37 °C, and the secretion of phospholipase, urease, and other extracellular components [2,3]. A common theme is that many of the virulence factors require transport to the plasma membrane or cell wall, or secretion to the extracellular environment. This is particularly true for the capsule polysaccharide that is considered to be the major virulence factor of the fungus [1,3,4]. The capsule is known to have a variety of immunomodulatory effects, and acapsular mutants are attenuated for virulence in animal models [3–7]. Many factors, such as iron starvation, serum, carbon dioxide levels, and pH and glucose levels, influence the size of the capsule in C. neoformans [4,8]. Protein trafficking is also important to localize the enzyme laccase to the cell wall for the polymerization of diphenol substrates to produce melanin [9]. Melanization in C. neoformans contributes to survival within alveolar macrophages, resistance to oxidative stress, and extra-pulmonary dissemination; melanin may also protect the fungus from environmental predators such as amoebae or from UV irradiation [10–15].

The cyclic adenosine 5′-monophosphate (cAMP)/protein kinase A (PKA) signaling pathway regulates capsule size, mating, melanin formation, and virulence in C. neoformans [16–19]. Several components of the pathway have been characterized, including the genes encoding a Gα protein (Gpa1), adenylyl cyclase (Cac1), a candidate G-protein–coupled receptor (Gpr4), phosphodiesterase (Pde2), and the catalytic (Pka1, Pka2) and regulatory (Pkr1) subunits of PKA. Expression of *GPA1* is induced by nitrogen limitation, and *gpa1* mutants show attenuated capsule production, reduced melanin formation, sterility, and lower virulence [16]. Adenylyl cyclase mutants display the same phenotypes as *gpa1* mutants [19]. Recently, Bahn et al. [20] identified Aca1, an adenylyl cyclase–associated protein, which functions in parallel with Gpa1 to control Cac1. The cAMP pathway is activated in part through Gpr4; interestingly, this receptor responds to amino acids and influences capsule but not melanin formation [21]. Xue et al. [21] speculated that a separate G-protein–coupled receptor and a hexose transporter could function upstream of the cAMP pathway to mediate the response to glucose in the context of melanin synthesis.

The genes encoding the catalytic *(PKA1, PKA2)* and regulatory subunits *(PKR1)* in C. neoformans have been disrupted in strains of both the A and D serotypes [17,18,20]. In a serotype A strain, the *pka1* mutant is sterile, unable to produce melanin or capsule, and is avirulent; these phenotypes resemble those of the *gpa1* and *cac1* mutants [16,19]. Mutants defective in *PKR1* may constitutively express PKA activity, independent of upstream signals. Disruption of *PKR1* suppresses the capsule and melanin defects of the *gpa1* mutant, causes cells to display an enlarged capsule phenotype, and results in hypervirulence [17]. In a serotype D strain, the Pka1 catalytic subunit is not required for mating, haploid fruiting, production of capsule or melanin, and virulence [17,18]. Instead, a second PKA catalytic subunit (Pka2), which is present in both serotype A and D strains, regulates mating, haploid fruiting, and virulence factor formation. Pka2 has no apparent role in mating and melanin production in the serotype A strain, indicating that differences exist for the roles of Pka1 and Pka2 in strains of the different serotypes [18,20].

Although components of the cAMP/PKA pathway have been identified, downstream targets have yet to be characterized in detail. A recent microarray experiment identified genes whose expression is dependent on Gpa1 and revealed that cAMP regulates multiple genes at the transcriptional level to control capsule and melanin production [22]. Importantly, the *LAC2* gene was identified by this approach, and this gene encodes a second laccase that is adjacent in the genome to the more thoroughly characterized laccase gene, *LAC1*. Like *LAC1, LAC2* transcript levels are induced by response to glucose deprivation [22].

The influence of the cAMP/PKA pathway on transcription has been investigated in several fungi, including *Saccharomyces cerevisiae, Candida albicans,* and Ustilago maydis [23–26]. Microarray analyses with mutants defective in each of the three catalytic subunits of PKA (Tpk1–3) in S. cerevisiae revealed that Tpk2 negatively regulates genes for iron uptake and positively regulates genes for trehalose degradation and water homeostasis [26]. Tpk1 influenced the expression of genes for branched chain amino acid biosynthesis. In a related approach, Jones et al. [25] used arrays to examine the influence of constitutive expression of the cAMP/PKA pathway due to loss of the *PDE2* gene encoding a cAMP phosphodiesterase. These results linked cAMP signaling to ribosome biogenesis and the response to stress including a connection with the unfolded protein response (UPR) pathway. Additionally, the expression of a number of genes encoding cell wall functions was altered in the mutant. In *C. albicans,* transcriptional profiling of a mutant defective in adenylyl cyclase linked cAMP signaling to ribosome biogenesis, metabolism, cell wall functions, the dimorphic transition, and the response to stress [23]. The influence on cell wall functions and the dimorphic transition was further confirmed through the analysis of mutants defective in pathway components. For example, deletion of the *PDE2* gene encoding a high affinity cAMP phosphodiesterase results in higher sensitivity to heat shock and agents that challenge cell wall integrity, perturbation of hyphal and pseudohyphal development, and changes in sensitivity to antifungal drugs [27,28]. Transcriptional analysis by serial analysis gene expression (SAGE) of U. maydis mutants defective in the catalytic or regulatory subunits of PKA also revealed connections with ribosome biogenesis, morphogenesis, and metabolism. In addition, this analysis revealed PKA regulation of phosphate acquisition and a role for phosphate sensing in morphogenesis [24].

In this report, we used SAGE to examine the transcriptomes of mutants defective in the catalytic *(pka1)* or the regulatory *(pkr1)* subunits of PKA and found that links between PKA, ribosome biogenesis, stress response, and metabolic functions are conserved in C. neoformans. However, we also observed PKA regulation of components of the secretory pathway. Coupled with the observed changes in transcript levels for ribosomal proteins and stress chaperones, these SAGE results suggested that PKA plays a role in remodeling secretion to facilitate cell surface expression of virulence factors. These observations are consistent with recent reports that a defect in a secretory component Arf1 reduces capsule size [29] and that exocytosis and specialized vesicles mediate the secretion of capsule polysaccharide [30,31]. In addition, we found that PKA activity had specific effects on genes for capsule production, indicating that the kinase may act both transcriptionally and post-transcriptionally to influence virulence. Functional analyses confirmed that PKA regulates the response to temperature stress and that secretion inhibitors block capsule production. Finally, deletion of a PKA-regulated gene, *OVA1,* revealed an epistatic relationship with *pka1* in the control of capsule size and melanin formation. This gene encodes a putative phosphatidylethanolamine-binding protein (PEBP) that may link phospholipid metabolism, secretion, and cAMP signaling in C. neoformans.

## Results

### Differential Expression of Virulence-Associated Functions in PKA Mutants

Given the phenotypes of mutants defective in the catalytic and regulatory subunits of PKA [17,18], we hypothesized that the cAMP/PKA pathway might regulate the expression of known (i.e., *CAP* and *CAS* genes [4]) and novel genes for capsule formation and other virulence factors at the transcriptional level. To test this idea, we generated SAGE libraries for the wild-type (WT) strain H99 (71,067 tags) and for the *pka1* and *pkr1* mutants (68,350 and 49,224 tags, respectively) using cells grown in low iron medium (LIM) to induce capsule expression (Table S1). The SAGE profiles were compared to identify differential transcript levels (*p* ≤ 0.01): 599 tags were found to be differentially expressed when the WT library was compared with the *pka1* library, 285 were differential between the WT and *pkr1* libraries, and 419 were differential between *pka1* and *pkr1* (Table S2). Overall, the percentages of differential tags between the libraries ranged from 1.31% (*pkr1* versus WT) to 2.64% (*pka1* versus WT), indicating substantial changes in the transcript profiles in the mutants. The 100 most abundant tags for each library are listed in Table S3, and a global comparison of shared unique tag sequences among the three libraries is presented in Figure S1. We annotated all of the differentially expressed tags with regard to the corresponding genes and then sorted the genes into functional categories. This analysis revealed that genes in several categories were affected by defects in PKA (Tables 1–4; Tables S3–S5). These genes encode known virulence proteins, ribosomal proteins and other components of the translation machinery, a large number of heat shock and stress proteins, protein trafficking components, cytoskeleton proteins, transporters, cell surface and extracellular proteins, and a variety of metabolic functions for phospholipid synthesis, the tricarboxylic acid cycle, and glycolysis. Overall, these observations reflect the conserved role of the cAMP pathway in coupling environmental sensing (e.g., of nutrient levels) with metabolism and growth.

**Table 1:**
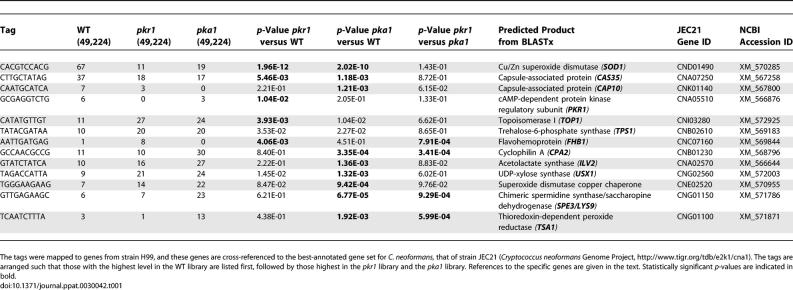
SAGE Tags for Genes Encoding Known Virulence Factors or Virulence-Associated Functions

We initially focused on categories containing known genes for capsule and melanin formation (*CAP* and *CAS* genes) or genes with known associations with virulence (e.g., response to oxidative stress). We found that tags for the capsule-related genes *CAS35* and *CAP10* [4] had reduced levels in the *pka1* and *pkr1* mutants compared with tags for the WT strain in the low iron condition (Table 1). Interestingly, a tag for the *USX1* gene encoding UDP-xylose synthase for capsule xylosylation was up-regulated in both mutants [32]. However, other capsule genes such as *CAP59, CAP60,* and *CAP64* (and other *CAS* genes) did not show significant expression in all libraries and thus could not be assessed for differential transcript levels. Although we did not detect tags for the *LAC1* and *LAC2* genes, we did find a tag for a putative multi-copper oxidase/ferro-O2-oxidoreductase gene (acidic laccase) at lower levels in both mutants, and we identified a putative copper transporter with a lower tag level in the *pka1* mutant (Table 3). Previously, Pukkila-Worley et al. [22] found that several genes for capsule production and two laccase genes, *LAC1* and *LAC2,* were regulated by the cAMP pathway component Gpa1. Laccase is a copper-dependent enzyme and there is evidence for a role for copper transport in influencing laccase activity [15,33].

Several other genes implicated in virulence, including genes for functions involved in oxidative, nitrosative, and temperature stress, showed differential tag levels in the PKA mutants. These included the *SOD1* (Cu/Zn superoxide dismutase) gene that had reduced tag levels in both mutants (Table 1). The antioxidant function of Sod1 is critical for the virulence of the fungus, and deletion of *SOD1* affects the expression of a number of virulence factors, including laccase, urease, and phospholipase [34–37]. Notably, a tag for the gene encoding a superoxide dismutase copper chaperone also showed a higher level in the mutants. Enzymes that counteract oxidative and nitrosative stress contribute to virulence in *C. neoformans.* In this context, we found that the tag for the *FHB1* gene encoding flavohemoprotein [38] had a higher abundance in the *pkr1* mutant library relative to the WT library. In addition, the *TSA1* gene encoding thiol peroxidase plays a role in resistance to oxidative and nitrosative stress and is also necessary for virulence (Table 1) [39]. Among genes known to be important for high-temperature growth, a tag for the *TPS1* gene for trehalose-6-phosphate synthase was more abundant in the *pkr1* and *pka1* mutants. Tps1 is involved in the synthesis of the stress protectant sugar, trehalose, and contributes to the ability of the fungus to grow at 37 °C [2, 40]. Amino acid metabolism genes such as *ILV2* (acetolactate synthase) and the *SPE3/LYS9* chimeric gene (chimeric spermidine synthase/saccharopine dehydrogenase) are also required for growth at elevated temperature and for full virulence [41,42]. Tags for both *ILV2* and *SPE3/LYS9* were more abundant in the *pka1* mutant libraries. Cyclophilin A is also required for growth at host temperature [43], and a tag for the corresponding gene had a higher abundance in the *pka1* mutant library. Cyclophilins have peptidyl-prolyl isomerase activity and catalyze protein folding [44]. Finally, a tag for *TOP1* (topoisomerase I) was elevated in the *pka1* and *pkr1* mutant libraries (Table 1). *TOP1* is essential in C. neoformans and its regulation under stress conditions may have an impact during initiation of infection [45].

Overall, these results indicate that PKA influences transcript levels for several functions implicated in the response to stress and in virulence. However, the patterns of regulation, with higher or lower transcript levels in one or both mutants, indicate a complex influence of PKA on virulence-related functions that likely involves direct and indirect control mechanisms. For example, 377 tags had higher levels and 507 had lower levels in both mutant libraries relative to the WT library (Table S2). Similar complex patterns of regulation were also observed in the SAGE analysis of PKA mutants defective in catalytic or regulatory subunits in U. maydis [24].

### Connections between PKA, Stress, and Secretion

**Figure 1:**
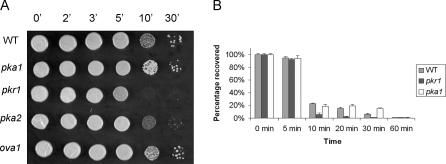
Comparison of Heat Shock Sensitivity for the WT Strain, and the *pka1* and *pkr1* Mutants (A) Cells (10^5^) were subjected to heat shock treatment at 50 °C for the indicated time and spotted on YPD plates. The plates were incubated for 3 d at 30 °C. (B) Enumeration of colony forming units (CFUs) for the strains after heat shock. The data are expressed as the percent of the starting number of cells recovered after heat shock and represent the mean values ± standard deviation (SD) from three independent experiments.

Our analysis of the SAGE data revealed that ∼20 tags representing stress response genes were elevated in the *pka1* mutant compared with those in the WT strain (Table 2). Some of these genes were also elevated in the *pkr1* mutant relative to WT, and six were down-regulated relative to the WT library. These tags represent genes with sequence similarity to a number of heat shock proteins (HSPs), including Hsp10, Hsp12, Hsp60, Hsp70, Hsp90, and Sks2. Coupled with the genes for other stress-responsive proteins, such as glutathione peroxidase, transaldolase, alpha-trehalose-phosphate synthase, thioredoxin-dependent peroxide reductase, and cyclophilin A, these results indicate a conserved connection between PKA and stress in C. neoformans. Many of these proteins are important for secretion as well as resistance to oxidative and nitrosative stress, and several have been linked to virulence in C. neoformans or other pathogens [36–39,43,46–48]. For example, glutathione peroxidases are important for defense against oxidative killing in bacterial pathogens such as Streptococcus pyogenes [49]. HSPs are produced in response to heat stress [50], and the SAGE results predicted that the *pka1* mutant would be more resistant to temperature and oxidative stress compared with the WT and *pkr1* strains because of the higher levels of tags associated with the HSPs. We confirmed this prediction by showing that the *pka1* mutant was more resistant to a 50 °C heat shock than the WT H99 strain and the *pkr1* mutant (Figure 1). The difference between the *pka1* mutant and the WT strain was particularly evident in the plate assay at 10 min of heat shock (Figure 1A) and at 30 min for the quantitative assay (Figure 1B, *p* < 0.01). The *pkr1* mutant was notably more sensitive to heat shock than either of the other strains (Figure 1). It is interesting to note that the higher transcript levels for genes for heat shock and oxidative stress proteins in the *pka1* mutant must not make a sufficient or appropriate contribution to virulence given the attenuation observed for this mutant [17].

**Table 2:**
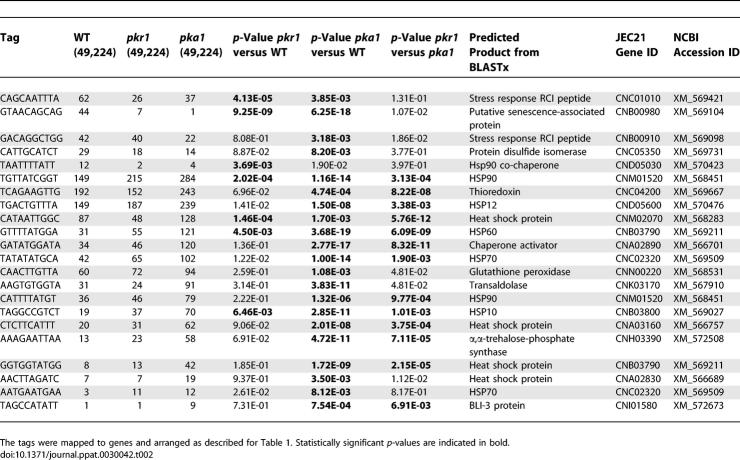
SAGE Tags for Genes Related to the Response to Stress

**Table 3:**
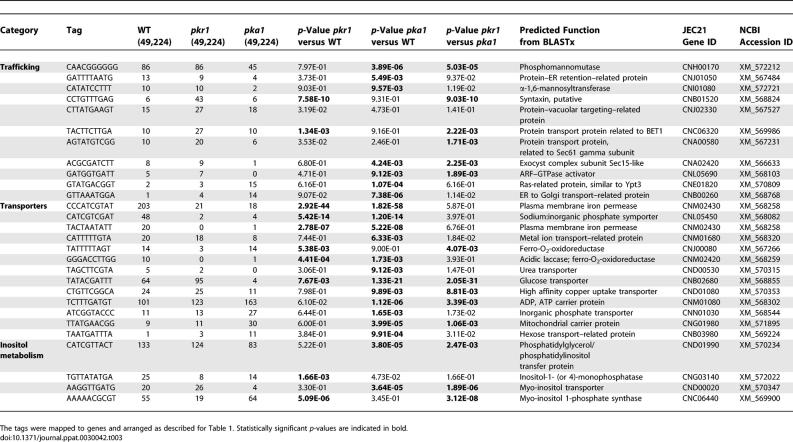
SAGE Tags for Genes Encoding Vesicle Trafficking Machinery, Transporters, and Proteins for Inositol Metabolism

**Table 4:**
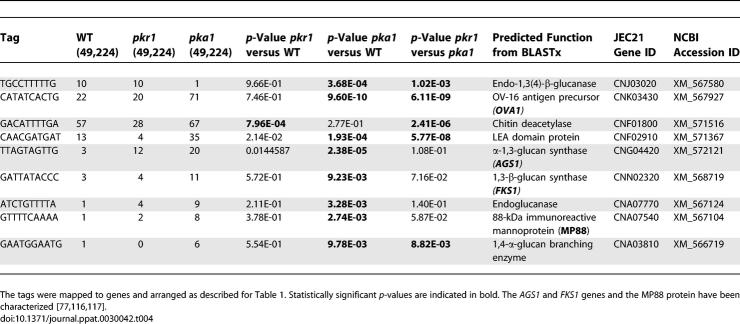
SAGE Tags for Genes Related to Cell Wall, Cell Surface, and Extracellular Proteins

Previously, Jones et al. [25] found that a mutation in *PDE2,* encoding a cAMP phosphodiesterase, influenced the expression of components of the UPR pathway in S. cerevisiae. The data for C. neoformans suggest that PKA activity influences ribosome biogenesis and the expression of stress-related proteins such as endoplasmic reticulum (ER) chaperone components that may have a role in protein folding. We therefore examined the functional categories of genes from the SAGE data for those known to be positively and negatively regulated by ER stress and UPR in yeast [51,52]. As listed in Tables 2 and 3, the tags for a number of predicted functions related to ER stress were influenced by the *pka1* and/or *pkr1* mutations, including myo-inositol phosphate synthase, protein disulfide isomerase, cyclophilin, and Hsp70 family member chaperones. Additionally, tags for genes in other functional categories related to the UPR were also influenced by the PKA mutations. These included genes for ribosomal proteins (Table S4) and amino acid biosynthetic enzymes (Table 1; Table S5). In mammalian cells, a hallmark of ER stress is a reduction in protein synthesis to reduce ER loading, and our SAGE data revealed that tags matching genes for 48 ribosomal protein genes were elevated in the *pkr1* library and/or reduced in the *pka1* library (Table S4). A similar influence of cAMP signaling on ribosomal protein expression has been previously described in other fungi [23–25,27,53]. These observations raise the possibility that one component of the differential influence of the *pka1* and *pkr1* mutations on capsule size might be due to an influence on the ER and the trafficking of proteins and capsule components. In this light, we noted that the SAGE data contained a group of tags elevated in the *pkr1* library and/or reduced in the *pka1* library that identified genes for predicted proteins connected with protein or vesicle trafficking machinery (Table 3). These functions included a putative membrane protein required for vesicular transport (Bet1), a syntaxin, an ADP-ribosylation factor (ARF) GTPase activator, a protein–ER retention–related protein, a subunit of the protein transport protein Sec61, a protein–vacuole targeting-related protein, a phosphomannomutase, and an exocyst complex subunit (rsec15). Two other tags identified a gene for a member of the Rab superfamily of Ras-related proteins (Ypt3) and a gene for an ER–Golgi transport–related protein; these were elevated in the *pka1* library relative to the *pkr1* and/or WT libraries. In yeast and animal cells, trafficking of cargo molecules through the secretory pathway relies on packaging and delivery of membrane vesicles [54]. Bet1 and syntaxin are SNAREs (soluble N-ethylmaleimide-sensitive factor attachment protein receptors) that mediate vesicle fusion with target membranes in S. cerevisiae. The ARF GTPase activator protein stimulates the nucleotide exchange and hydrolysis reactions of ARF GTPases and therefore regulates Arf-mediated vesicle transport [55,56]. Disruption of the *C. neoformans ARF1* gene encoding an ADP ribosylating factor has recently been shown to reduce capsule size [29].

**Figure 2:**
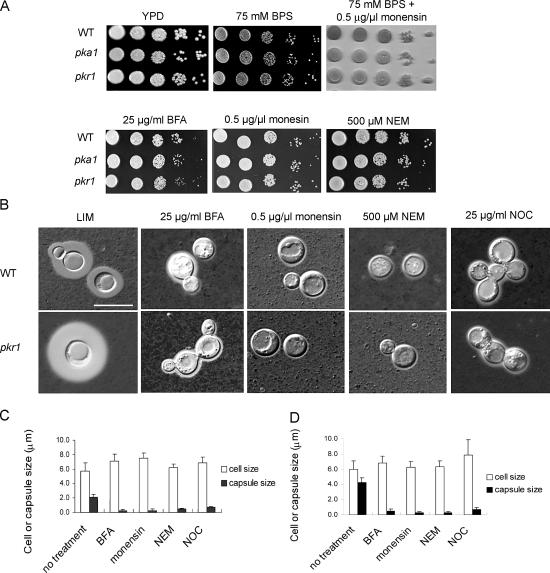
Suppression of Capsule Formation by Secretion Inhibitors (A) Sensitivity of the WT strain H99 and the *pka1* and *pkr1* mutants to trafficking inhibitors. Photographs of cells on medium containing the ferrous iron chelator BPS, and the chelator plus monensin, are included to demonstrate growth of the strains under low iron conditions similar to those used in the broth cultures (B) to measure capsule size. (B) Suppression of capsule formation in both the WT strain and the *pkr1* mutant after treatment with trafficking inhibitors at the indicated concentrations. Cells were cultured in LIM at 30 °C and examined at 72 h after addition of the inhibitors indicated. Chemicals and concentrations are indicated at the top and the strains are noted on the left of the photographs. Bar = 10 μM. (C and D) Sixty cells from the WT (H99) strain (C) and the *pkr1* mutant (D) were measured to determine cell diameter and capsule radius with and without treatment with the trafficking inhibitors (as shown in [B]). The capsule sizes of the treated cells were statistically different (*p* <0.001) from the sizes for the untreated cells in all cases. Each bar represents the average of 60 measurements.

Capsule elaboration must involve the trafficking of a large amount of polysaccharide material along with the proteins necessary for assembly to the cell surface [30,31]. Based on the SAGE data, we hypothesized that PKA could influence capsule formation by regulating secretion. We therefore examined the effects of known vesicle trafficking inhibitors that target functions identified by the SAGE data on cell growth, morphology, and capsule formation. These inhibitors included brefeldin A (BFA), nocodazole (NOC), monensin, and N-ethylmaleimide (NEM). BFA is known to arrest the anterograde transport of proteins between the ER and Golgi apparatus by interfering with the action of ARF. As mentioned, the SAGE data revealed that a gene for a putative ARF–GTPase-activating protein (GAP) was regulated by PKA. NOC inhibits vesicle trafficking by interfering with microtubule formation, and we were prompted to test this compound because the transcript levels for α and β tubulin were elevated in the *pka1* mutant (Table S5). Monensin is a Na+/H+ ionophore that blocks intracellular transport in both the trans-Golgi and post-Golgi compartments. NEM is a cysteine alkylating agent that interferes with disulfide bond formation. The inhibitors did not block growth when tested with cells on yeast extract peptone dextrose (YPD) plates for 3 d (for NOC, cells were grown in either liquid YPD or low iron medium), although the cells grew slower in the presence of NEM or BFA (Figure 2A; unpublished data). The WT and mutant strains also grew in the presence of the iron chelator bathophenanthroline disulfonic acid (BPS) with or without the addition of monensin (Figure 2A). These conditions were tested because LIM was employed to assess capsule formation. In this regard, capsule size was significantly reduced (*p* < 0.001) when the cells were grown at different concentrations of the inhibitors in LIM at 30 °C (Figure 2B–2D). This result was found both for WT, and for the *pkr1* mutant that otherwise displays an enlarged capsule. Additionally, an abnormal morphology was observed for cells of both strains in LIM with 10 or 25 μg/ml of NOC in that mother and daughter cells failed to separate (Figure 2B). The influence on capsule size was more pronounced at higher concentrations of inhibitors and with longer treatment for both strains (48 h). The SAGE data and the inhibition assays together support a model in which connections between PKA and the secretory pathway explain, at least in part, why the loss of PKA activity results in a small capsule [17].

A key aspect of trafficking is the biosynthesis of phospholipids to support membrane formation for the ER, Golgi, and vesicular network [57]. Inositol is an essential component of phosphospholipids as well as phosphoinositides that act as second messengers in signaling cascades and that regulate membrane trafficking [58–60]. Additionally, *INO1* encoding inositol 1-phosphate synthase is a well-characterized target of both UPR and PKA regulation in S. cerevisiae [61,62]. Genes related to inositol metabolism were identified by SAGE including the C. neoformans ortholog of *INO1* encoding myo-inositol 1-phosphate synthase; the tag for this gene had a reduced level in the *pkr1* mutant (Table 3). A tag for a gene encoding a putative inositol-1- (or 4)-monophosphatase was found to have lower levels in the *pka1* and *pkr1* mutants relative to WT. This enzyme is important for converting glucose 6-phosphate to myo-inositol phosphate. A tag for a gene encoding phosphatidylglycerol/phosphatidylinositol transfer protein (PITP) was elevated in both the *pkr1* and WT libraries relative to the *pka1* library. PITP was named due to its ability to transport phosphatidylinositol between membrane compartments, and recent data indicate that PITP plays an important role in the coupling of PIP2 (phosphatidylinositol-4,5-bisphosphate, a substrate for phospholipase C) to signaling and membrane trafficking in mammalian cells [63,64]. In *S. cerevisiae,* PITP is required for cell viability [63,64], and in *Yarrowia lipolytica,* PITP is required for differentiation from the yeast to the mycelial growth form [63]. The SAGE data also identified a tag for an ortholog of the *S. cerevisiae ITR1* gene that encodes a myo-inositol transporter protein, the major permease for inositol uptake [65]. Taken together, the data indicate that PKA regulates genes whose products influence the level of free inositol in the cell, and inositol is known to play a major role in the regulation of phospholipid biosynthesis [66,67].

**Figure 3:**
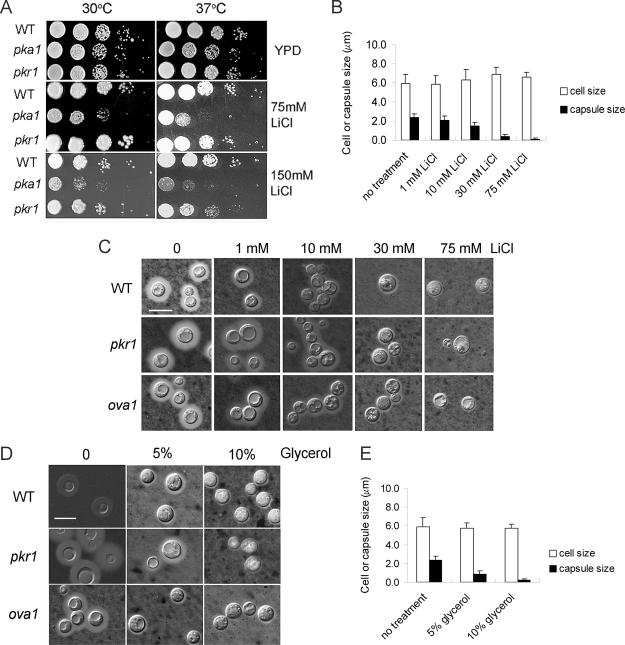
Lithium Chloride and Glycerol Treatments Influence Capsule Size (A) Sensitivity of the WT strain H99 and the *pka1* and *pkr1* mutants to growth on YPD medium containing LiCl. The strains are listed on the left side of the panel. The plates were incubated at 30 °C or 37 °C for 5 d before being photographed. (B) Sixty cells from the WT (H99) strain treated with LiCl at the indicated concentrations were measured for cell diameter and capsule radius. Each bar represents the average of measurements for 60 cells. The capsule sizes of the cells treated with 10, 30, and 75 mM LiCl were statistically different (*p* <0.001) from the sizes for the untreated cells. (C) Examination of capsule formation in the WT strain, and the *pkr1* and *ova1* mutants, in a range of LiCl concentrations. Bar = 10 μM. (D) Capsule formation in the WT and the *pkr1* and *ova1* mutants in the presence of glycerol at two concentrations (5% and 10%). Bar = 10 μM. In (B–D), cells were cultured in LIM at 30 °C for 16 to 72 h with addition of glycerol or LiCl as indicated. (E) Sixty cells from the WT (H99) strain grown with the indicated concentrations glycerol were measured for cell diameter and capsule radius. Each bar represents the average of 60 measurements. The capsule sizes of the cells with either concentration of glycerol were statistically different (*p* <0.001) from the sizes for the untreated cells.

To functionally examine the role of inositol metabolism (and phospholipid biosynthesis indirectly) in the control of capsule production, we tested the influence of lithium on growth and capsule size. Lithium is an inhibitor of inositol monophosphatase and may also influence inositol uptake as well as other cellular processes [68,69]. We found that the *pka1* mutant was more sensitive to 75 mM and 150 mM lithium chloride (LiCl), especially at 37 °C, compared with the *pkr1* mutant and the WT strain (Figure 3A). The sensitivity of the *pka1* mutant is consistent with reduced transcript levels for genes encoding a myo-inositol transporter and the inositol monophosphatase in the mutant relative to the WT strain. Changes in transcript levels for these genes were also observed in the *pkr1* mutant (Table 3), and we noted slightly reduced growth for this mutant in 150 mM LiCl at 37 °C. Given the capsule defect in the *pka1* mutant [17] and the influence of hypertonic salt solutions on capsule size [70], we reasoned that LiCl might also influence capsule formation in WT cells and the *pkr1* mutant, and this was the case. As shown in Figure 3B and 3C, treatment with a range of relatively low LiCl concentrations (1 mM to 75 mM) reduced capsule size in cells grown in LIM in a dose-dependent manner. This result supports the idea that inositol and perhaps phospholipid metabolism are important for trafficking capsule material, although it is also possible that lithium influences other processes in C. neoformans.

Growth in the presence of glycerol is also known to influence phospholipid metabolism, and glycerol can act as a chemical chaperone to influence protein trafficking indirectly by mediating protein folding and transport [71,72]. We therefore examined the influence of glycerol in the WT and mutant cells and found inhibition of capsule formation (Figure 3D and 3E). This result further supports the hypothesis that phospholipid biosynthesis and vesicle trafficking are important for capsule formation.

### Expression of Genes Encoding Transporters, Cell Wall Components, and Putative Extracellular Proteins

Several differential tags identified genes encoding proteins targeted to intracellular organelles, the plasma membrane, or the cell surface. For example, 13 of these tags matched genes encoding membrane-associated transporters (Table 3). These included transporters related to carbohydrate import/export such as two monosaccharide transporters (hexose transport-related protein, glucose transporter) and the myo-inositol transporter that are elevated in *pkr1* library and/or reduced in *pka1* library (Table 3). A tag for hexose transporter gene was also elevated in the *pka1* library relative to the WT and *pkr1* libraries. The transport and sensing of sugars may be a critical component of both the cAMP signaling pathway and the acquisition of substrate to support capsule formation. For example, genes for glucose transporters were expressed during cryptococcal experimental meningitis and interaction of the fungus with macrophages [73,74], and a hexose transporter has been proposed to be upstream of the cAMP pathway in C. neoformans [21]. In addition, one putative hexose transporter has been functionally characterized and found to be involved in capsule formation, but not virulence, in a Caenorhabditis elegans killing test [75]. Other tags identified genes for a group of transporters related to phosphate uptake and assimilation. A transcript for a phosphate transporter was elevated in the *pka1* library, and a tag for a gene encoding putative sodium:inorganic phosphate symporter was reduced in both the *pka1* and *pkr1* libraries compared with the WT library. These observations are similar to our description of an influence of PKA on the expression of phosphate acquisition and storage functions in U. maydis [24,76]. Additional tags in this group identified a gene for a urea transporter that was elevated in the WT library and a tag for a metal ion transport–related protein that was lower in the *pka1* library.

**Figure 4:**
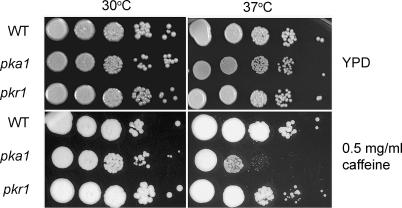
Sensitivity of PKA Mutants to Caffeine Ten-fold serial dilutions of the WT and the *pka1* and *pkr1* mutants were grown on YPD medium containing caffeine at 30 °C and 37 °C. The strains used for all treatments are listed on the left side of the panel. The plates were incubated for 5 d before being photographed.

Transcripts encoding proteins involved in cell wall synthesis and having an extracellular location were also affected in the *pka1* or *pkr1* mutants (Table 4). In this category, eight tags were elevated and one tag was reduced in the *pka1* mutant. Some of the corresponding genes encode a chitin deacetylase, an 88-kDa mannoprotein (MP88), and a polypeptide with similarity to the OV-16 antigen presursor that we designated Ova1 [77]. MP88 may be relevant to virulence because this mannoprotein is known to stimulate T-cell responses [77]. Other tags represented genes for putative enzymes involved in cell wall synthesis or remodeling; these enzymes included an α-1,3-glucan synthase (Ags1), a β-1,3-glucan synthase (Fks1), an endoglucanase, an α-1,4 glucan branching enzyme, and an endo-1,3(4)-β glucanase. The influence of PKA on candidate genes for cell wall functions prompted an examination of the sensitivity of the strains to agents that challenge cell wall integrity such as SDS, Congo red, calcofluor white, and caffeine. Significant differences between the WT strain and the *pka1* and *pkr1* mutants were not found except for caffeine, where the *pka1* mutant grew more slowly than the WT strain and the *pkr1* mutant at 37 °C (Figure 4, and unpublished data). Caffeine affects many cellular processes, including cAMP phosphodiesterase activity, and has been used to detect cell integrity phenotypes in *S. cerevisiae,* especially in the context of the mitogen-activated protein kinase/cell wall integrity pathway [78]*.* The increased sensitivity of the *pka1* mutant to caffeine supports the idea that cAMP signaling is involved in the maintenance of cell wall integrity in *C. neoformans,* although additional influences of caffeine cannot be ruled out. We also examined the sensitivity of strains to osmotic stress and did not observe differences on medium with 1.5 M NaCl, 1.5 M KCl, or 1.5 M sorbitol (unpublished data).

### The PKA-Regulated Gene *OVA1* Is Epistatic to *pka1* for Capsule and Melanin Formation

**Figure 5:**
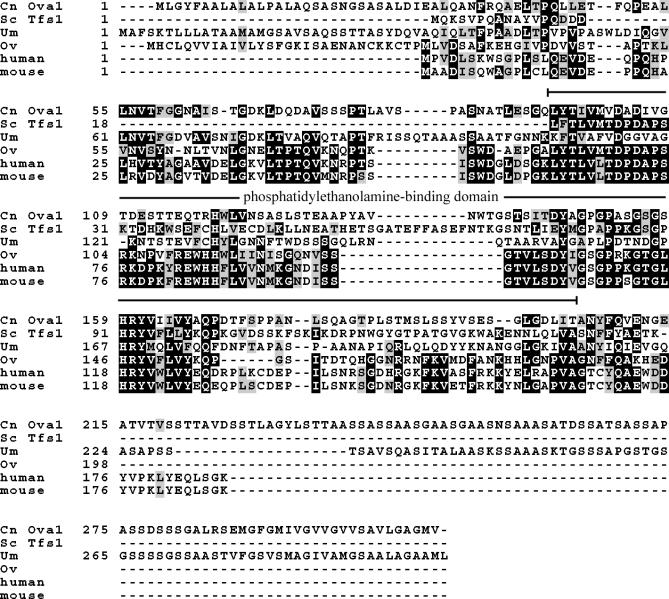
Multiple Alignment of Ova1 with TFS1 and Other PEBP Family Members The amino acid sequence alignment was performed with Clustal W (http://www.ch.embnet.org); identical residues are boxed in black and similar residues are shown in gray. The phosphatidylethanolamine-binding domain is indicated by a bold arrow spanning residues 91–204 of Ova1. Cn, C. neoformans var. *grubii;* Ov, *Onchocerca volvulus;* Sc, Saccharomyces cerevisiae; Um, Ustilago maydis.

The discovery of a connection between secretion, PKA, and capsule formation in C. neoformans suggests that specific downstream targets of PKA may have regulatory roles that influence capsule size. As indicated earlier, we identified a gene, *OVA1,* with an elevated tag count in the *pka1* mutant, indicating that PKA has a negative influence on the transcription of the gene*. OVA1* is also of particular interest because the tag was abundant in a SAGE library prepared with cells from the cerebral spinal fluid of infected rabbits [74], and the gene encodes a predicted polypeptide with similarity to the conserved PEBP family present in many organisms such as mammals, fungi, worms, and bacteria (Figure 5) [79–82]. Ova1 also shows similarity to the OV-16 antigen of the river blindness parasite Onchocerca volvulus and was identified as a mannoprotein in C. neoformans by Huang et al. [77], indicating that the protein is secreted. In *S. cerevisiae,* the most similar protein (a yeast PEBP), Tfs1 (for “twenty-five suppressor”), was isolated as multicopy suppressor of the *cdc25–1* mutant [82]. Cdc25 in yeast is one of the Ras guanine exchange factors (GEFs) that activates Ras to subsequently stimulate adenylyl cyclase. Tfs1 also inhibits Ira2, a Ras GAP in yeast, thus making an additional connection with the cAMP pathway. Furthermore, Tfs1 is an inhibitor of carboxypeptidase Y, a well-characterized cargo protein for examining trafficking in *S. cerevisiae.* Overall, these observations suggest a conserved connection between putative PEPB proteins and cAMP signaling in fungi, and lead us to hypothesize that Ova1 functions in the secretory pathway to influence trafficking functions for capsule formation.

**Figure 6:**
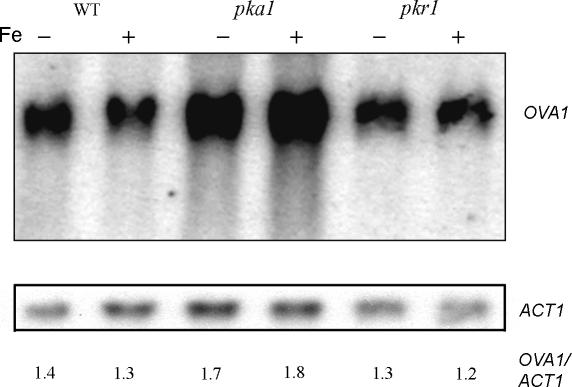
Northern Analysis of *OVA1* Expression in WT and Mutant Strains in Media with Two Different Levels of Iron Cells were grown in LIM (designated −) and LIM + Fe (designated +). The Northern blot was prepared from total RNA and hybridized with a DNA fragment from the *OVA1* gene. The same filter was hybridized with a DNA fragment from the actin gene *(ACT1)* as a loading control. Note that the hybridization pattern is consistent with the SAGE data, and also indicates that iron does not significantly influence *OVA1* transcript levels in any of the strains. The numbers at the bottom indicate the ratio of the hybridization signals for the *OVA1* and *ACT1* genes, as determined from the scanned images. The expression of *OVA1* was also confirmed by a quantitative real-time PCR analysis (Figure S2).

**Figure 7:**
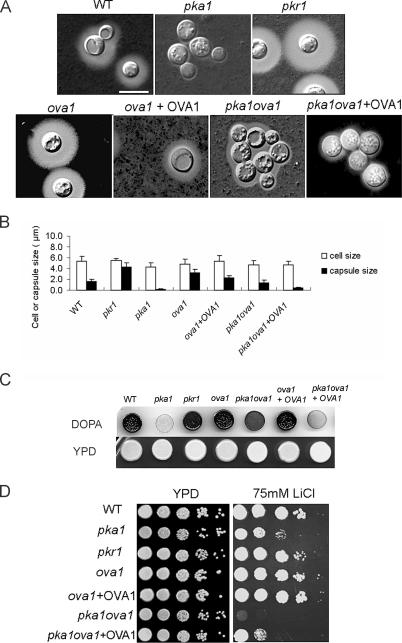
Influence of the *ova1* Mutation on Capsule and Melanin Formation, and Epistasis with *pka1* (A) Capsule size is shown by staining with India ink for the WT and mutant strains. Cells were cultured either in liquid LIM or on DMEM plates (for capsule induction) at 30 °C. The photographs are of cells cultured for 48 h in LIM, and the same results were obtained in several independent trials. Bar = 10 μM. (B) Sixty cells from the cultures used for (A) were measured for cell diameter and capsule radius. Each bar represents the average of 60 measurements. The capsule sizes were statistically different (*p* <0.001) for all pair-wise comparisons except WT versus *pka1ova1,* and *pka1* versus *pka1ova1+OVA1.* (C) Melanin formation was tested in the strains indicated by inoculation of cultures on L-DOPA or YPD plates (as a control to demonstrate equal growth) and incubation for 2 d at 30 °C. (D) Sensitivity of the *pka1* mutant and the *pka1 ova1* double mutant to LiCl. Cells were washed and 10-fold serial dilutions of a 10^6^ cells/ml suspension were plated on either YPD or YPD + 75 mM LiCl.

We initially confirmed that *OVA1* shows a higher transcript level in the *pka1* mutant compared with the WT strain and the *pkr1* mutant by RNA blot analysis (Figure 6) and real-time PCR analysis (Figure S2). Given that the SAGE libraries were prepared with cells from LIM, we also examined the influence of iron on *OVA1* transcript levels and found no effect (Figure 6). We subsequently generated deletion mutants for *OVA1* as well as reconstituted strains in which *OVA1* was reintroduced to complement the mutation. The *ova1* deletion mutants did not show significant growth differences at 30 °C or 37 °C compared to WT, but did display a slightly enlarged capsule in different inducing media, including LIM (Figure 7A and 7B). We included the *ova1* mutant in our tests of the influence of LiCl and glycerol on capsule formation and found that the mutant was as sensitive as the WT strain and the *pkr1* mutant (Figure 3C and 3D). The *OVA1* gene was also disrupted in the *pka1* mutant background to examine epistasis with regard to capsule and melanin formation. Interestingly, the *pka1 ova1* double mutant showed partial restoration of capsule formation compared to the *pka1* mutant in both LIM and Dulbecco's modified Eagle's medium (DMEM), suggesting that Ova1 functions downstream of PKA to influence capsule size (Figure 7A and 7B). The double mutant additionally showed partial restoration of melanin production compared to the *pka1* mutant on medium containing dopamine as a substrate (Figure 7C). We also tested the *pka1 ova1* mutant for sensitivity to lithium and found enhanced sensitivity compared to either of the single mutants (Figure 7D). The WT or the *pka1* phenotypes were restored upon reintroduction of the *OVA1* gene into the *ova1* mutant or the *pka1 ova1* double mutant, respectively. Given the putative phosphatidylethanolamine-binding domain, the lithium sensitivity of the double mutant, and the influence of PKA on transcript levels for genes in inositol metabolism, we propose that Ova1 plays a negative role in phospholipid-related trafficking functions needed for capsule production and potentially influences laccase transport. Of course, it is also possible that Ova1 functions in other processes to influence capsule size and melanin production in the background of the *pka1* mutation. Finally, we performed a preliminary test of the role of Ova1 in virulence because of our observations that *ova1* mutants had a slightly enlarged capsule. We inoculated the mutant, the WT, and the complemented strain into mice, and we found no defect in the ability of the mutant to cause a lethal infection (unpublished data).

## Discussion

The cAMP pathway regulates a variety of processes in fungi, including nutrient sensing, growth, the response to stress, and morphogenesis [83, 84]. For *C. neoformans,* Alspaugh et al. [16] and D'Souza et al. [17] showed that this pathway controls formation of the polysaccharide capsule that is the major virulence factor of the fungus. We therefore examined the influence of mutations in genes encoding a catalytic subunit or the regulatory subunit of PKA on the transcriptomes of cells from capsule-inducing medium to gain insight into the mechanisms underlying changes in capsule phenotype. We found that defects in PKA had effects on transcript levels for genes involved in virulence, ribosome biogenesis, the response to stress, vesicle (protein) trafficking, membrane transport, and cell wall biogenesis. Although some of these genes represent conserved targets of cAMP signaling in fungi, our analysis also revealed a novel relationship between cAMP signaling and the secretory pathway in C. neoformans with coordinated changes in ribosomal protein and heat shock gene expression. This discovery focused our attention on PKA-regulated secretion as a potential central component of virulence factor elaboration. In particular, transcriptional changes in the *pka1* and *pkr1* mutants indicated an influence at several stages in the secretory pathway, including translocation (Sec61 and Hsp70/Kar2), maturation in the ER (Hsp70/Kar2, protein disulfide isomerase), vesicle formation and fusion (Bet1, syntaxin), Golgi transport (α-1,6-mannosyltransferase, phosphatidylglycerol/phophatidylinositol trans-fer protein), and vesicle delivery to the plasma membrane (e.g., Ypt3). Support for a key role of PKA in the regulation of secretion also came from observed transcriptional changes for genes encoding chaperones and enzymes for phospholipid metabolism. Treatment with inhibitors of protein secretion and chemicals that influence phospholipid metabolism (lithium and glycerol) confirmed a role in capsule elaboration. These results may partially explain observations in the literature such as the finding of Jacobson et al. [70] that a high salt concentration suppresses capsule size; salt stress is known to influence phospholipid metabolism and the accumulation of compatible solutes such as glycerol in yeast [85].

The influence of defects in PKA on the transcriptome in C. neoformans suggests a model in which PKA regulates the expression of secretory pathway components to control the elaboration of virulence factors at the cell surface. In particular, we believe that changes in the regulation of PKA activity, as would likely be found in the *pkr1* mutant, could mediate a remodeling of the secretory pathway to accommodate the delivery of large amounts of polysaccharide material. Additionally, PKA could directly influence the activity or localization of transcription factors that control the transcript levels of some of the genes detected by SAGE, for example, in response to nutrient availability (e.g., glucose, nitrogen, iron, phosphate). A paradigm for this scenario exists with the influence of PKA on the Opi1p repressor of inositol synthesis in yeast [86]. In *C. neoformans,* phosphorylation by PKA could also positively or negatively influence the activity of proteins in the secretory pathway, and this may influence a UPR-like response to indirectly regulate transcription factors leading to the observed transcriptional responses. In addition to transcriptional effects, a more direct influence is also possible because PKA has been shown to phosphorylate Sso exocytic t-SNAREs to inhibit SNARE assembly and vesicle fusion in S. cerevisiae [87,88]. Furthermore, PKA has been shown to phosphorylate secretory components such as cysteine string proteins (CSPs) (DNAj co-chaperone), Snapin, Rim1, Snap-25, syntaphilin, and synapsin in higher eukaryotes [89]. The cAMP/PKA pathway also regulates aspects of autophagy, a process linked to the UPR in yeast [90]. In general, the UPR pathway in S. cerevisiae provides a useful paradigm for the connection between molecular events in the ER that regulate secretion and that signal to the nucleus to cause transcriptional changes [51,52]. The UPR results in a transcriptional influence on ∼400 genes: regulated functions include ER chaperones, phospholipid biosythesis, ER-associated protein degradation (ERAD), cell wall components, and anti-oxidative stress proteins; hallmark target genes in yeast include *INO1, KAR2,* and *PDI1* [51, 52]. We observed transcriptional changes in similar functions in the PKA mutants for C. neoformans. For example, genes involved in the response to oxidative stress and heat shock, and genes for chaperones and ribosomal proteins represented some of the largest groups that were differentially transcribed in the mutants. We also noted differential transcripts for functions associated with ERAD such as genes for putative ubiquitin ligases that showed elevated transcripts in the *pka1* library (G. Hu, unpublished data). The variety of potential levels of regulation will make it challenging to establish the mechanisms for specific targets of PKA control in C. neoformans.

There is a growing body of evidence to link capsule synthesis and secretion in C. neoformans. Capsule biosynthesis may take place in the ER and the Golgi as suggested by subcellular studies that identified “wall vesicles” as potential structures involved in capsule biosynthesis and/or secretion [91–97]. Alternatively, capsule synthesis could occur at the cell membrane [91–97]. Walton et al. [29] showed that disruption of the *ARF1* gene, which encodes the ARF GTPase involved in vesicle transport, reduced capsule size. In addition, Yoneda and Doering [30] found that mutation of a *SEC4/RAB8* homolog resulted in the accumulation of vesicles containing material that stained with anti-capsular antibody. These authors concluded that capsule material is synthesized internally and secreted by exocytosis. More recently, Rodrigues et al. [31] showed that C. neoformans cells in culture and from infected macrophages produce extracellular vesicles that contain capsule polysaccharide. These vesicles are believed to provide a mechanism for moving high molecular weight capsule material across the cell wall. Two capsule-associated gene products, Cap10 and Cap60, are localized to intracellular vesicles and to a compartment adjacent to the nuclear envelope, respectively [5–7]. Another capsule-associated gene, *CAP59,* may also participate in polysaccharide export [94]. Connections between membrane trafficking and virulence in C. neoformans have also been established by Erickson et al. [98]. They characterized the *VPH1* gene encoding a vacuolar (H+)-ATPase and found that disruption of this gene results in defects in capsule formation, laccase production, urease production, and growth at 37 °C. A role in vesicular acidification was proposed to contribute to trafficking of capsule polysaccharide and laccase, with additional potential roles in signal sequence processing or glycosylation. Secretion would also potentially influence melanin production by influencing the localization of laccase to the cell wall.

Based on the SAGE results, we searched the set of PKA-regulated genes for those that might play a role in secretion and that might have a connection with cAMP signaling in other organisms. One gene, *OVA1,* encoded a predicted extracellular mannoprotein [77] with similarity to PEBPs and to the Tfs1p protein of S. cerevisiae [82]. PEBPs play several roles in mammalian cells, including lipid binding, inhibition of serine proteases, and regulation of signaling components such as heterotrimeric G proteins, as well as other functions [99]. Lipid (e.g., oxysterol) binding proteins are known to play a role in vesicle transport [100], and PKA could potentially regulate vesicle transport through an influence on these proteins. In this context, interconnections with phospholipid metabolism are possible because, as mentioned above, the transcription factor Opi1p acts as a lipid sensor and is a target of PKA phosphorylation [101]. The similarity of *OVA1* to *TFS1* is interesting because of a shared connection with PKA. Specifically, Tfs1p was initially identified as a multicopy suppressor of the cdc25–1 mutation in S. cerevisiae [82]. More recent work has shown that Tfs1p is an inhibitor of the Ras-GAP encoded by *IRA2* and carboxypeptidase Y (CPY is a well-characterized protein that traffics through the yeast secretory pathway) [102,103]. Given that *CDC25* encodes the Ras-GEF and *IRA2* encodes the Ras-GAP, and that Ras functions in S. cerevisiae to stimulate cAMP signaling, Tfs1p appears to be an activator of the RAS/cAMP/PKA pathway that establishes links to lipid binding and potential secretion functions. *TFS1* is also overexpressed in response to oxidative stress, diauxic shift, and heat shock, and contains a stress response element (STRE), indicating transcriptional control by Msn2/Msn4 [102]. Furthermore, loss of Tfs1 suppresses growth inhibition caused by caffeine [102]. The functional similarities between Tfs1 and Ova1 and the shared PEBP domain suggested that Ova1 might play a role in linking PKA and secretion in C. neoformans. The SAGE data support this idea because the *OVA1* transcript was found to be elevated in the *pka1* mutant along with the heat shock/stress gene transcripts suggesting that *OVA1* might also be stress-responsive like *TFS1*. Deletion of *OVA1* partially restored capsule and melanin formation in a *pka1* mutant indicating Ova1 functions downstream of PKA and has a negative influence on capsule size and melanin accumulation. These observations suggest the hypothesis that Ova1 plays a regulatory role in the trafficking of protein and polysaccharide to the cell surface, perhaps as a component of secretory vesicles. Ova1 is predicted to carry a glycosylphosphatidylinositol (GPI) anchor that may serve to attach the protein to either the cell membrane or β-1,6-glucans in the cell wall. Although we did not observe significant defects in cell wall integrity in the *ova1* mutant (unpublished data), it is known that cAMP signaling is required in S. cerevisiae and C. albicans for maintenance of cell wall integrity [23,28,104]. There may be issues of redundancy to consider because another gene *(OVA2)* that is predicted to encode a PEBP is present in the C. neoformans genome.

In summary, a common theme in pathogenesis is the elaboration of extracellular and cell surface–associated virulence factors by pathogens. We propose that the cAMP pathway is critical for coordinating nutrient sensing with secretion in *C. neoformans,* particularly during infection. The SAGE analysis provides target genes that will be valuable for investigating the expression of secretory system components and virulence factors in the context of cryptococcal growth in mammalian hosts. Importantly, many of the genes that we have identified for secretion, heat shock, and transport showed abundant messages in the same strain of C. neoformans isolated from the cerebral spinal fluid of a rabbit model of cryptococcal meningitis [74]. These observations provide confidence that the in vitro conditions used for SAGE analysis of virulence factor regulation have relevance to infection. We should note that our SAGE analysis provides a view of the regulation of gene expression by PKA only in the serotype A background of C. neoformans. It is known that differences exist in PKA signaling between strains of the A and D serotypes [18]. Therefore, additional work is needed to explore whether the regulatory properties that we discovered are generally applicable to serotype D strains and other pathogenic *Cryptococcus* species. Finally, a more detailed understanding of functions that regulate capsule formation in C. neoformans may ultimately contribute to improved therapy. The capsule is an important therapeutic target because of its central role in virulence; in particular, accumulation of polysaccharide in the cerebral spinal fluid of patients is thought to be a contributing factor in the development of elevated intracranial pressure that results in neurological symptoms during cryptococcal meningitis [105,106].

## Materials and Methods

### Strains and media.

The C. neoformans var. *grubii* strain H99 (WT) and the derived mutants with defects in *PKA1, PKA2,* or *PKR1* [17] were generously provided by J. Heitman (Duke University). For SAGE library construction, cells were grown for 3 d at 30 °C on YPD (1% yeast extract, 2% Bacto peptone, 2% dextrose) plates from frozen stocks. A single colony was used to inoculate 5 mL of LIM prepared as described previously [107]. These cultures were grown overnight at 37 °C, and the cells (H99, 3.0 × 10^6^ CFU/mL; *pka1,* 3.0 × 10^6^ CFU/mL; *pkr1,* 1.2 × 10^6^ CFU/mL) were washed with sterile distilled water and inoculated into 45 mL of LIM for subsequent growth for 6 h at 37 °C. This time point was chosen to be consistent with previous SAGE experiments that identified iron-responsive genes [107]. Cells (H99, 5.0 × 10^7^ CFU/mL; *pka1,* 2.0 × 10^7^ CFU/mL; *pkr1,* 3.0 × 10^7^ CFU/mL) were harvested by centrifugation and flash frozen in an ethanol dry-ice bath before lyophilization overnight at −80 °C. L-DOPA medium was prepared as described [17].

### SAGE library construction.

RNA was isolated from lyophilized cells by vortexing with glass beads (3.0 mm, acid-washed and RNase-free) for 15 min in 15 ml of TRIZOL extraction buffer (Invitrogen, http://www.invitrogen.com). The mixture was incubated for 15 min at room temperature, total RNA was isolated according to the manufacturer's instructions (Invitrogen), and RNA quality was assessed by agarose gel electrophoresis. Total RNA was used directly for SAGE library construction as described by Velculescu et al. [108] using the I-SAGE kit (Invitrogen). The tagging enzyme for cDNA digestion was NlaIII, and 29 PCR cycles were performed to amplify the ditags during library construction. Colonies were screened by PCR (M13F and M13R primers) to assess the average clone insert size and the percentage of recombinants. Clones from the libraries were sequenced by BigDye primer cycle sequencing on an ABI PRISM 3700 DNA analyzer (AME Bioscience, http://www.amebioscience.com). Sequence chromatograms were processed using PHRED [109,110], and vector sequence was detected using Cross_match [111]. Fourteen-base-pair tags were extracted from the vector-clipped sequence, and an overall quality score for each tag was derived based on the cumulative PHRED score. Duplicate ditags and linker sequences were removed as described previously [107,108]. Only tags with a predicted accuracy of ≥99% were used in this study, and statistical differences between tag abundance in different libraries were determined using the methods of Audic and Claverie [112].

### SAGE data analysis.

An overview of the abundance classes for the three libraries is presented in Table S1. The number of different tag sequences and the total numbers of tags present in each abundance class for the WT, *pka1,* and *pkr1* strains are indicated. For preliminary assignment of tags to genes, we used the EST database available for strain H99 at the University of Okalahoma's Advanced Center for Genome Technology (http://www.genome.ou.edu/cneo.html). When an EST sequence could not be identified for a particular tag, we used the genomic sequence available for H99 at the Duke University Center for Genome Technology (http://cneo.genetics.duke.edu/data/index.html) and the Broad Institute (http://www.broad.mit.edu/cgi-bin/annotation/fungi/cryptococcus_neoformans) to identify contigs with unambiguous tag assignments. Of the 599 unique tag sequences found to have differential abundance between the WT, *pka1,* and *pkr1* libraries at a threshold *p*-value of less than 0.01 (Table S1), 25 were found to match two or more different locations in the genome sequence and were not included for further analysis. An additional 76 tags did not match any of sequences in either the genomic or EST databases for strain H99. These tags may result from reverse transcription or sequencing errors, incomplete H99 genomic or EST sequence data, or tag overlap of an intron position. The remaining tags could be unambiguously assigned to candidate transcripts, and the corresponding EST or genomic sequence was used to search the nonredundant database at the National Center for Biotechnology Information (NCBI) using BLASTx (Basic Local Alignment Search Tool). Each BLASTx result was inspected individually and recorded to prepare tables of tags and the corresponding predicted genes. In the case of genomic DNA sequences where introns are present, the expected values recorded were higher than would have been expected if the introns had been removed. The same was true for the results determined for both genomic sequences and ESTs compared to those that would have been expected if the sequences had been translated and the BLASTp algorithum were employed rather than a BLASTx algorithm.

### Phenotypic analysis.

To examine the response of C. neoformans to stress, exponentially growing cultures were washed, resuspended in H_2_O, and adjusted to 10^6^ cells/ml. The cell suspensions were the diluted 10-fold serially, spotted onto YPD medium supplemented with or without 1.5M KCl, 1.5M NaCl, 75mM, or 150mM LiCl, 0.5 mg/ml caffeine, 0.01% and 0.1% SDS, 0.5 or 1 mg/ml calcofluor white (Fluorescent Brighter 28), 1.5 M sorbitol, or 0.5 mg/ml Congo red. Plates were incubated for 3–4 d at 30 °C or 37 °C, and photographed. For heat shock treatment, early log phase cells grown at 30 °C were adjusted with YPD to 10^5^ cells/ml, and incubated in a 50 °C water bath for 0, 2, 3, 5, 10, 30, or 60 min, and 4 μl of cells were spotted onto YPD plates. The plates were incubated at 30 °C and growth was monitored for 3 d. Quantitative analysis was performed by plating cell dilutions to determine colony forming units.

### Construction of the *ova1::NEO* null allele.

An *ova1::NEO* disruption allele was constructed using the following primers and a modified overlap PCR procedure [113,114]. Briefly, the primers hug2–1/hug2–3 (CACGTGCCAAGACTGAACAT/ AGCTCACATCCTCGCAGCAGACAAGGGAGGGTCAAGG) and hug2–4/hug2–6 (TAGTTTCTACATCTCTTCCTCACAACGGAAAGGACGAT/ TATTCGCGGCTATTTGGAAC) were used with genomic DNA to obtain the left (∼1 kb) and right (∼1 kb) arms for the disruption construct. The selectable marker NEO was amplified using the primers hug2–2/hug2–5 (CCTTGACCCTCCCTTGTCTGCTGCGAGGATGTGAGCT/ ATCGTCCTTTCCGTTGTGAGGAAGAGATGTAGAAACTA) and the plasmid pJAF1 (J. Heitman), which contains the neomycin antibiotic resistance marker cassette for *C. neoformans.* The *ova1::NEO* allele results in the deletion of the complete open reading frame of *OVA1* (∼1.5 kb). The resulting 3.5-kb PCR product was used to transform strain H99 by biolistic transformation. Transformants were screened by colony PCR for the *ova1::NEO* allele using primers hug2-IntF/hug2-IntR (negative screen) (GCTCACAACACCGACAAC/GGAGACTTGATCACTGCGA and hug2–9/hug-NEO (CCAGCGATGCCATTTCCAT/AGCTCACATCCTCGCAGC) (positive screen). Primer hug2–9 was designed from the region upstream of *OVA1* and hug-NEO was designed for the NEO gene. Transformants in which the WT allele was replaced were confirmed by Southern blot hybridization. Three mutants containing the allele designated *ova1Δ* were studied further.

### Complementation of the *ova1Δ* mutant.

The *OVA1* gene for complementation of the *ova1Δ* mutant was amplified by PCR using primers ova1-BamH1–5 (CAGGGATCCAACCGTTCCATCAGGATGAC) and ova1-BamH1–3 (AGAGGATCCGCCAAGCGGTTCAATATAAGG), and H99 genomic DNA. The ∼3.35-kb product was digested with BamHI and cloned into the BamHI site of pCH233, creating plasmid pHG100. Strains ova1Δ-47 and pka1Δova1Δ–2 were transformed with pHG100 by biolistic transformation [107], and reintroduction of *OVA1* was confirmed by colony PCR and Southern blot analysis (unpublished data).

### Northern blot analysis and quantitative real-time PCR.

Total RNA was isolated as presented above and hybridization was performed as previously described [107]. The hybridization probe was prepared with a PCR-amplified DNA fragment of *OVA1* using the specific primers hug2-IntF and hug2-IntR, and lableled with ^32^P using an Oligolabelling kit (Amersham Biosciences, http://www.amershambiosciences.com). Scanned images were analyzed using an AlphaImager 3400 (Alpha Innotech, http://www.alphainnotech.com). Real-time PCR analysis was conducted using primers targeted to 3′ ends of the transcripts; primers were designed using PrimerExpress (Applied Biosystems, http://www.appliedbiosystems.com). Total RNA from the frozen cells was extracted as described above, DNA was removed by treatment with DNase I for 30 min at 25 °C, and cDNA was synthesized using random hexamers and Superscript transcriptase II (Invitrogen Canada). The resulting cDNA was used for real-time PCR with Power SYBR Green PCR mix (Applied Biosystems) according to the manufacturer's recommendations. An Applied Biosystems 7500 Fast Real-Time PCR System was used to detect and quantify the PCR products using the following conditions: incubation at 95 °C for 10 min followed by 40 cycles of 95 °C for 15 sec, and 60 °C for 1 min. The cDNAs of the ACT1 and GPD genes were used to normalize the data (Table S6; [22,73]). Dissociation analysis on all PCR reactions confirmed the amplification of a single product for each primer pair and the lack of primer dimerization (Applied Biosystems). Primers used for each gene are listed in Table S6. Relative gene expression was quantified using SDS software 1.3.1 (Applied Biosystems).

### Capsule formation.

A single colony from solid YPD medium for each strain was cultured overnight at 30 °C in liquid YPD medium. Two inducing media (LIM and agar-based DMEM) [107,115] were used to examine capsule formation. For DMEM, 3 × 10^5^ cells were spotted onto the plate, and incubated for 72 h at 30 °C. For LIM, an overnight culture in liquid YPD medium was harvested and diluted in low iron water, and 10^6^ cells were added into 3 ml of liquid LIM for further incubation at 30 °C for 48 h. After incubation, the capsule was stained by India ink and examined by differential interference microscopy (DIC).

### Inhibitor assays.

A single colony on solid YPD medium for each strain was cultured overnight at 30 °C in liquid YPD medium. Cells were collected and washed with H_2_O, and 10^6^ cells were added into 3 ml of liquid LIM containing different concentrations of vesicle trafficking inhibitors (as indicated) and further cultured in a shaking incubator at 30 °C. Cells were examined at different time points (16, 48, and 72 h) by DIC after staining with India ink. Vesicle trafficking inhibitors included BFA, NOC, monensin, and NEM. LIM was also generated by the addition of BPS to 75 mM. All chemicals were obtained from Sigma (http://www.sigmaaldrich.com). Statistical analysis of capsule size was performed using Student's *t*-test.

## Supporting Information

Figure S1 Venn Diagram of the Relationships between the SAGE Tags for the WT Strain and the cAMP Signaling Mutants The three libraries share 4,609 tag sequences. The pair-wise comparisons of the libraries revealed the following numbers of shared tag sequences: *pka1* and *pkr1,* 5,648 tag sequences; *pkr1* and WT, 6,133 tag sequences; *pka1* and WT, 6,528 tag sequences. This analysis also revealed that the *pkr1* mutant library was 97.2% similar to the WT library in terms of the overall gene expression pattern relative to a 95.3% similarity between the *pka1* and WT libraries. The *pka1* and *pkr1* libraries had 95.6% similarity. The numbers in parentheses for each library indicate the total number of tag different sequences in each library.
(30 KB TIF)

Figure S2 Relative Quantification of Gene Expression of Selected Transcripts in the WT Strain and the *pka1* and *pkr1* Mutants (A) Quantitative real-time PCR was used to analyze the expression of eight selected genes found by SAGE to be differentially expressed in the WT and mutant strains. Similar results were obtained with either *ACT1* or *GPD1* as the control transcript for normalization. The real-time PCR analysis was repeated with three independent samples for each strain, and each bar represents the average of three independent measurements. The gene designations for the orthologs in the JEC21 genome are indicated below the graph, and the primer sequences for amplification are given in Table S6.
(B) Levels of SAGE tags for the genes in the WT and mutant strains are shown for comparison with the PCR analysis. Note that the trends in the patterns of gene expression are consistent between the two methods, but the fold changes are different, perhaps due to the differences in sensitivity for the two methods.
(67 KB TIF)

Table S1 Analysis of SAGE Libraries (45 KB DOC)

Table S2 Number of Differentially Expressed Tags in Each SAGE Library (22 KB DOC)

Table S3 One Hundred Most Abundant Tags in Each SAGE Library (186 KB DOC)

Table S4 Tags for Ribosome Biogenesis Genes and Related Functions (87 KB DOC)

Table S5 Tags for Genes Related to Carbohydrate and Amino Acid Metabolism, and Cytoskeleton and Vacuolar Function (102 KB DOC)

Table S6 Primer Sequences Used in Real-Time PCR Analysis (24 KB DOC)

### Accession Numbers

The GenBank (http://www.ncbi.nlm.nih.gov) accession numbers for the PEBP proteins discussed in this paper are human (P30086); mouse (AF300422_1); Onchocerca volvulus (P31729); Saccharomyces cerevisiae (CAA44015.1); and Ustilago maydis (XP_756473). The accession numbers for the *ACT1* and *GPD1* genes are XP_566845 and XP_571627, respectively. The sequence for *C. neoformans* var *grubii* (CNAG_02001.1 [homologue in *C. neoformans* var. *neoformans* JEC21, CNK03430]) is from the Broad Institute database for *C. neoformans* var. *grubii* (http://www.broad.mit.edu/annotation/genome/cryptococcus_neoformans/GeneIndex.html).

## Abbreviations

ARF: ADP-ribosylation factor
BFA: Brefeldin A
BPS: bathophenanthroline disulfonic acid
cAMP: cyclic adenosine 5′-monophosphate
CFU: colony forming unit
DMEM: Dulbecco's modified Eagle's medium
ER: endoplasmic reticulum
GAP: GTPase-activating protein
HSP: heat shock protein
LiCl: lithium chloride
LIM: low iron medium
NEM: N-ethylmaleimide
NOC: nocodazole
PEBP: phosphatidylethanolamine-binding protein
PITP: phosphatidylglycerol/phosphatidylinositol transfer protein
PKA: protein kinase A
SAGE: serial analysis gene expression
UPR: unfolded protein response
WT: wild-type
YPD: yeast extract peptone dextrose
