# Supplementary material for: Transcriptional Regulation by Protein Kinase A in Cryptococcus neoformans
Source: PLoS Pathog. 2007 Mar 16;3(3):e42. doi: 10.1371/journal.ppat.0030042 (PMC1828699; doi:10.1371/journal.ppat.0030042)
Supplement: Supplementary file 1 [file ppat.0030042.st001.doc]

| Supplemental Table S1. Analysis of SAGE libraries | | | | | | |
| --- | --- | --- | --- | --- | --- | --- |
|  | | | | | | |
| Tag families | WT | | *pkr1* | | *pka1* | |
|  | Tag sequence count  (%) | Total number  of tags  (%) | Tag sequence count  (%) | Total number  of tags  (%) | Tag sequence count  (%) | Total number  of tags  (%) |
|  |  |  |  |  |  |  |
| Singletons | 9732  (60.9) | 9732  (13.7) | 7291  (61.6) | 7291  (14.8) | 7941  (59.8) | 7941  (11.6) |
|  |  |  |  |  |  |  |
| 2 to 4 | 3706  (23.2) | 9655  (13.6) | 2777  (23.5) | 7158  (14.5) | 3017  (22.7) | 7899  (11.6) |
|  |  |  |  |  |  |  |
| 5 to 9 | 1308  (8.2) | 8563  (12.0) | 938  (7.9) | 6128  (12.4) | 1085  (8.2) | 7123  (10.4) |
|  |  |  |  |  |  |  |
| 10 to 99 | 1143  (7.2) | 26115  (36.7) | 760  (6.4) | 16875  (34.3) | 1135  (8.6) | 27873  (40.5) |
|  |  |  |  |  |  |  |
| ≥ 100 | 89  (0.55) | 17002  (23.9) | 64  (0.5) | 11772  (23.9) | 92  (0.69) | 17514  (25.6) |
|  |  |  |  |  |  |  |
| Total | 15798 | 71067 | 11830 | 49224 | 13270 | 68350 |
|  |  |  |  |  |  |  |
| Each tag sequence has a 99% probability of being correct based on PHRED analysis (109, 110) | | | | | | |
