# Supplementary material for: Transcriptional Regulation by Protein Kinase A in Cryptococcus neoformans
Source: PLoS Pathog. 2007 Mar 16;3(3):e42. doi: 10.1371/journal.ppat.0030042 (PMC1828699; doi:10.1371/journal.ppat.0030042)
Supplement: Supplementary file 2 [file ppat.0030042.st002.doc]

| Supplemental Table S2. Number of differentially expressed tags in each SAGE library | | | |
| --- | --- | --- | --- |
|  | | | |
| Differential tags | Library comparisons | | |
|  | *pka1* vs. WT | *pkr1* vs. WT | *pkr1* vs. *pka1* |
|  |  |  |  |
| Total | 599 | 285 | 419 |
| Up-regulated | 262^a^ | 115^a^ | 234 |
| Down-regulated | 337^b^ | 170^b^ | 185 |

Differential expression was evaluated at the 99% confidence level using the methods of Audic and Claverie (112). The terms up-regulated and down-regulated refer to the first library in each comparison. ^a^ These tags represent a total of 307 tags that are elevated in both mutant libraries relative to WT. ^b^ These tags represent a total of 507 tags that are reduced in both mutant libraries relative to WT.
