# Supplementary material for: Transcriptional Regulation by Protein Kinase A in Cryptococcus neoformans
Source: PLoS Pathog. 2007 Mar 16;3(3):e42. doi: 10.1371/journal.ppat.0030042 (PMC1828699; doi:10.1371/journal.ppat.0030042)
Supplement: Supplementary file 3 [file ppat.0030042.st003.doc]

| Supplemental Table S3. One hundred most abundant tags in each SAGE library | | | | |
| --- | --- | --- | --- | --- |
|  |  |  |  | |
| Tags | WT (49224) | Predicted product from BLASTx | JEC21 gene ID | NCBI Accession ID |
| AAAAAAAAAA* | 645 | multiple hits |  |  |
| GCATTCTTTA | 368 | ribosomal protein s11 | CNA06500 | XM_566991 |
| ACTACGTTCT | 283 | ribosomal protein p1 | CNA06350 | XM_566980 |
| ACATCGATCT | 281 | ribosomal protein L31 | CNA06820 | XM_566913 |
| GTATCAACGG | 247 | Predicted protein | unknown |  |
| GCATTTCTTT | 245 | NADH dehydrogenase (ubiquinone) | CNA01370 | XM_567227 |
| GAAAAAAAAA | 232 | no hit |  |  |
| AGTACTCTTC | 215 | ribosomal protein P2 | CNF02150 | XM_571539 |
| ATTGAGATGG | 213 | no hit |  |  |
| CTTTGGATCA | 204 | ribosomal protein L30/L30A | CNA02210 | XM_567145 |
| CCCATCGTAT | 203 | plasma membrane iron permease | CNM02430 | XM_568258 |
| ACATTTCCGA | 202 | putative ribosomal protein L14B | CNJ02260 | XM_567521 |
| CACTTGGATC | 201 | ribosomal protein L23 | CNK03100 | XM_567903 |
| GTCGTAGAGT | 192 | enolase | CNC00160 | XM_569379 |
| TCAGAAGTTG | 192 | thioredoxin | CNC04200 | XM_569667 |
| GCAGATCTAT | 189 | ribosomal protein RPL39 | CND05700 | XM_570480 |
| TAGTGTCCCG | 173 | 6-phosphogluconate dehydrogenase | CNK01050 | XM_567793 |
| ATAAAAAAAA | 168 | no hit |  |  |
| TATGATAGTG | 160 | ribosomal protein L7a (L8) | CNH02040 | XM_572378 |
| GATGCTTTTT | 160 | ribosomal protein L19B | CNI01090 | XM_572717 |
| CACATTCTTT | 157 | ubiquitin/S27a fusion protein | CND03060 | XM_570192 |
| CATTGTGACT | 157 | ribosomal protein S7 | CNI01120 | XM_572711 |
| GCAAAAAAAA | 151 | no hit |  |  |
| TGACTGTTTA | 149 | heat shock protein 12 | CND05600 | XM_570476 |
| TGTTATCGGT | 149 | heat shock protein 90 homolog | CNM01520 | XM_568451 |
| TAACCCAAAT | 149 | ribosomal protein L7 | CNA06360 | XM_566981 |
| CGACAGACCG | 144 | translation elongation factor 1 (TEF1) | CNM01300 | XM_568462 |
| TATACCTATG | 134 | ribosomal protein s3ae (S1) | CNB04880 | XM_568980 |
| CATCGTTACT | 133 | phosphatidylglycerol/phosphatidylinositol  transfer protein | CND01990 | XM_568462 |
| CATTGAGGTT | 132 | ribosomal protein S22 | CNK02900 | XM_567885 |
| CTCAGCGATG | 130 | cytokine inducing-glycoprotein | CNC01660 | XM_569968 |
| GCATTGGCGT | 128 | hypothetical protein | CNC01620 | XM_569506 |
| AGAGATTTCT | 125 | translation elongation factor 2 (TEF2) | CND06220 | XM_570549 |
| AGCATATAAA | 125 | ribosomal protein L9 | CNA00250 | XM_566460 |
| GGCCGACCTG | 121 | ribosomal protein L1 | CNJ03370 | XM_567416 |
| ATCCCGTGGC | 121 | ribosomal protein S14 | CNF00670 | XM_571241 |
| GCTTCACAGA | 119 | ribosomal protein L12 | CNC06990 | XM_569829 |
| CACGGCGCAT | 117 | unknown | unknown |  |
| AACCTTGCAT | 116 | ribosomal protein L20A | CNJ01560 | XM_567335 |
| ACTATCGCCT | 116 | ubiquitin conjugating enzyme UBC1 | CNF02760 | XM_571364 |
| GCCGTTCTTA | 116 | predicted protein | unknown |  |
| TATGATTTTA | 114 | ribosomal protein S28 | CNG04360 | XM_572112 |
| ATTCAAAAAA | 112 | hypothetical protein | CNJ00800 | XM_567460 |
| ATGAAAAAAA | 109 | ubiquitin transferase | CNM01820 | XM_568304 |
| ACAAAAAAAA | 109 | multiple hits | unknown |  |
| Supplemental Table S3. cont. |  |  |  |  |
| CATCTTTCCT | 108 | ribosomal protein P0 | CNG00300 | XM_571764 |
| GGTTTGTACA | 108 | probable ribosomal protein S29 | CNC04290 | XM_570001 |
| CTATTGGTCG | 108 | ribosomal protein S8 | CNG03720 | XM_572078 |
| CACCACATTC | 106 | ribosomal protein L18 | CND03460 | XM_570672 |
| CACATCGTGC | 105 | mitochondrial isocitrate dehydrogenase1 | CNJ02060 | XM_567378 |
| CAGAGATGTG | 102 | nonhistone protein 6 | CNF04730 | XM_571615 |
| TCGTCTGAAG | 102 | ribosomal protein L5B | CNC05360 | XM_569733 |
| TCTTTGATGT | 101 | ADP, ATP carrier protein | CNM01080 | XM_568302 |
| GTCTCTTTAC | 100 | ribosomal protein L10A | CNE03920 | XM_571019 |
| ACACGTCTGG | 100 | ATP synthase G chain, mitochondrial | CNC03840 | XM_569622 |
| GGTCAGTCGA | 100 | hypothetical protein | CNJ00800 | XM_567460 |
| TATTTCAGTT | 99 | histone H3 | CNJ02540 | XM_567545 |
| AAGCGATTTT | 96 | ribosomal protein S26 | CNH01770 | XM_572351 |
| AGAACTCAAA | 94 | ribosomal protein S6B | CND02780 | XM_570222 |
| TCTCTTCCGT | 94 | carboxypeptidase | CND00600 | XM_570309 |
| GTTACATACA | 93 | cytokine inducing-glycoprotein | CNC01660 | XM_569968 |
| GCCTTGTGCC | 90 | predicted protein | unknown |  |
| TATTCATAAC | 89 | ribosomal protein L37a | CNC00700 | XM_569366 |
| GAATAGTGGG | 89 | hypothetical protein | CNA07830 | XM_567130 |
| AGGATGAGAA | 88 | hypothetical protein | CNC00760 | XM_569376 |
| CATAATTGGC | 87 | putative heat shock protein | CNM02070 | XM_568283 |
| TATTACAGCT | 87 | ribosomal protein L6 | CNE03060 | XM_571207 |
| CAACGGGGGG | 86 | phosphomannomutase | CNH00170 | XM_572212 |
| CTCTCTATGT | 86 | ribosomal protein S24 | CND04480 | XM_570598 |
| TATATGTGTA | 85 | heat shock protein 12 | CNG04220 | XM_572088 |
| TGATGGAAGC | 85 | p21 protein (wos2) | CNK01080 | XM_567796 |
| CCAATGACGT | 84 | GTP-binding nuclear protein Spi1p | CNE02890 | XM_571000 |
| GAATAATAGA | 83 | predicted protein | unknown |  |
| TACGATTGCG | 83 | translation initiation factor eIF-5A | CND05400 | XM_570147 |
| TAAAAAAAAA | 82 | no hit |  |  |
| GAGAACAGAA | 80 | hypothetical protein | unknown |  |
| AAAGAAGCCC | 80 | no hit |  |  |
| GATGCACCTG | 78 | probable guanosine-diphosphatase | CNB04810 | XM_568971 |
| TTACACAGCA | 78 | putative ribosomal protein S19 | CNC06100 | XM_569872 |
| TGGATGAGAT | 73 | ribosomal protein S3 | CNA01060 | XM_566546 |
| TAAATGGTGC | 72 | ribosomal protein RPL22 | CND05910 | XM_570540 |
| AAGGGTGGTG | 71 | ribosomal protein L24 | CNG02920 | XM_571980 |
| GCTTCCACGA | 71 | ribosomal protein L23A | CNC00350 | XM_569415 |
| TTAGTTGTGT | 70 | predicted protein | unknown |  |
| AAATGGTTTG | 70 | no hit |  |  |
| AACGGTTATG | 69 | ribosomal protein L3 | CNK02240 | XM_567698 |
| CTTGTAGAAA | 69 | no hit |  |  |
| TAACGCATAA | 69 | hypothetical protein | CNM00840 | XM_568420 |
| ACTAAAAAAA | 68 | no hit |  |  |
| TACTTGACCA | 68 | UDP-N-acetylglucosamine diphosphorylase | CNF01520 | XM_571302 |
| CACGTCCACG | 67 | Cu/Zn superoxide dismutase | CND01490 | XM_570285 |
| CAGGTCCGTG | 66 | progesterone binding protein | CNM01220 | XM_568281 |
| Supplemental Table S3. cont |  |  |  |  |
| TGAAAAAAAA | 65 | no hit |  |  |
| GACTTTTTTA | 64 | glycoprotein, putative | CNM00090 | XM_568387 |
| TATACGATTT | 64 | monosaccharide transporter | CNB02680 | XM_568855 |
| CAATCTTGTG | 63 | probable ATP-dependent RNA helicase DBP2  (p68-like protein) | CNF01240 | XM_571269 |
| CATTCCTTCA | 63 | hypothetical protein | CNA02520 | XM_566638 |
| GACATATGAA | 62 | alpha-amylase | CNH03660 | XM_572528 |
| ACTATCACAA | 62 | ribosomal protein L27A | CNA07570 | XM_567107 |
| CAGATCTTCT | 61 | guanine nucleotide-binding protein | CNH01270 | XM_572317 |
|  |  |  |  |  |
| Tags | *pkr1* (49224) | Predicted product from BLASTx | JEC21 gene ID | NCBI Accession ID |
| ACTACGTTCT | 603 | acidic ribosomal protein p1 | CNA06350 | XM_566980 |
| AGTACTCTTC | 525 | ribosomal protein P2 | CNF02150 | XM_571539 |
| GCATTCTTTA | 523 | ribosomal protein s11 | CNA06500 | XM_566991 |
| ACATCGATCT | 403 | ribosomal protein L31 | CNA06820 | XM_566913 |
| GCAGATCTAT | 384 | ribosomal protein RPL39 | CND05700 | XM_570480 |
| TATGATAGTG | 346 | ribosomal protein L7a (L8) | CNH02040 | XM_572378 |
| AAAAAAAAAA* | 325 | multiple hits |  |  |
| TATACCTATG | 303 | ribosomal protein s3ae (S1) | CNB04880 | XM_568980 |
| CACTTGGATC | 258 | ribosomal protein L23 | CNK03100 | XM_567903 |
| AACCTTGCAT | 253 | ribosomal protein L20A | CNJ01560 | XM_567335 |
| CACCACATTC | 251 | ribosomal protein L18 | CND03460 | XM_570672 |
| CTTTGGATCA | 243 | ribosomal protein L30/L30A | CNA02210 | XM_567145 |
| GGTTTGTACA | 225 | ribosomal protein S29 | CNC04290 | XM_570001 |
| TTACACAGCA | 225 | ribosomal protein S19 | CNC06100 | XM_569872 |
| TGTTATCGGT | 215 | heat shock protein 90 homolog | CNM01520 | XM_568451 |
| ATTGAGATGG | 214 | no hit |  |  |
| TAACCCAAAT | 208 | ribosomal protein L7 | CNA06360 | XM_566981 |
| ACATTTCCGA | 202 | ribosomal protein L14B | CNJ02260 | XM_567521 |
| TGACTGTTTA | 187 | heat shock protein 12 | CND05600 | XM_570476 |
| GTCTCTTTAC | 186 | ribosomal protein L10A | CNE03920 | XM_571019 |
| TATTCATAAC | 184 | ribosomal protein L37a | CNC00700 | XM_569366 |
| GTCGTAGAGT | 173 | enolase | CNC00160 | XM_569379 |
| CACATTCTTT | 165 | ubiquitin/S27a fusion protein [imported] | CND03060 | XM_570192 |
| TCGTCTGAAG | 161 | ribosomal protein L5B | CNC05360 | XM_569733 |
| GATGCTCTGT | 161 | ribosomal Protein RPL39 | CND05700 | XM_570480 |
| ACACGTCTGG | 159 | ATP synthase G chain, mitochondrial | CNC03840 | XM_569622 |
| GCTTCACAGA | 155 | ribosomal protein L12 | CNC06990 | XM_569829 |
| TCAGAAGTTG | 152 | thioredoxin | CNC04200 | XM_569667 |
| CATTGAGGTT | 147 | ribosomal protein S22 | CNK02900 | XM_567885 |
| ACTATCACAA | 147 | ribosomal protein L27A | CNA07570 | XM_567107 |
| GAAAAAAAAA | 144 | no hit |  |  |
| CACATTGATA | 140 | ribosomal protein L13A | CNM02320 | XM_568413 |
| AATGGATTAA | 140 | senescence-associated protein | CNJ00800 | XM_567460 |
| TGGATGGGCA | 139 | ubiquinol-cytochrome c oxidoreductase  subunit VIII | CNC02900 | XM_569987 |
| CATCTTTCCT | 137 | ribosomal protein P0 | CNG00300 | XM_571764 |
| CTCTCTATGT | 136 | ribosomal protein S24 | CND04480 | XM_570598 |
| Supplemental Table S3. cont. |  |  |  |  |
| CATTGTGACT | 135 | ribosomal protein S7 | CNI01120 | XM_572711 |
| GATGCTTTTT | 133 | ribosomal protein L19B | CNI01090 | XM_572717 |
| CATTCCTTCA | 128 | hypothetical protein | CNA02520 | XM_566638 |
| TATGATTTTA | 125 | ribosomal protein S28 | CNG04360 | XM_572112 |
| TGATGGAAGC | 124 | P21 protein, wos2 | CNK01080 | XM_567796 |
| AGAACTCAAA | 124 | ribosomal protein S6B | CND02780 | XM_570222 |
| CATCGTTACT | 124 | phosphatidylglycerol/phosphatidylinositol  transfer protein | CND01990 | XM_570234 |
| TCTTTGATGT | 123 | ADP, ATP carrier protein | CNM01080 | XM_568302 |
| ATCCCGTGGC | 123 | ribosomal protein S14 | CNF00670 | XM_571241 |
| TAAATGGTGC | 120 | ribosomal protein RPL22 | CND05910 | XM_570540 |
| ATAAGCTTTC | 120 | mannitol-1-phosphate dehydrogenase | CNG00600 | XM_571772 |
| CATACAGGTC | 119 | glutamine synthetase | CNA04370 | XM_566794 |
| GGTATTCTAT | 118 | ubiquinol-cytochrome-c reductase subunit IX | CNH02740 | XM_572447 |
| TATTACAGCT | 116 | ribosomal protein L6 | CNE03060 | XM_571207 |
| ACTATCGCCT | 115 | ubiquitin conjugating enzyme UBC1 | CNF02760 | XM_571364 |
| AAAACAAACT | 115 | cytochrome-c oxidase | CNF01340 | XM_571555 |
| TAGTGTCCCG | 114 | 6-phosphogluconate dehydrogenase | CNK01050 | XM_567793 |
| CTATTGGTCG | 113 | ribosomal protein S8 | CNG03720 | XM_572078 |
| CACGGCGCAT | 112 | unknown | unknown |  |
| AAGCGATTTT | 111 | ribosomal protein S26 | CNH01770 | XM_572351 |
| GCAAAAAAAA | 110 | no hit |  |  |
| CATTCGCATA | 109 | expressed protein | CNN00340 | XM_568554 |
| CGACAGACCG | 107 | Translation elongation factor 1 (TEF1) | CNM01300 | XM_568462 |
| GCTTCCACGA | 107 | ribosomal protein L23A | CNC00350 | XM_569415 |
| AGAGATTTCT | 105 | translation elongation factor 2 (TEF2) | CND06220 | XM_570549 |
| GATGCACCTG | 102 | guanosine-diphosphatase | CNB04810 | XM_568971 |
| CAGAGATGTG | 101 | nonhistone protein 6 | CNF04730 | XM_571615 |
| TGGATGAGAT | 100 | ribosomal protein S3 | CNA01060 | XM_566546 |
| AACGGTTATG | 96 | ribosomal protein L3 | CNK02240 | XM_567698 |
| TATACGATTT | 95 | monosaccharide transporter | CNB02680 | XM_568855 |
| GCATTGGCGT | 91 | hypothetical protein | CNC01620 | XM_569506 |
| AAATGGTTTG | 88 | no hit |  |  |
| ATAAAAAAAA | 87 | no hit |  |  |
| CAACGGGGGG | 86 | phosphomannomutase | CNH00170 | XM_572212 |
| GAATAGTGGG | 85 | hypothetical protein | CNA07830 | XM_567130 |
| ACAAAAAAAA | 85 | multiple hits | unknown |  |
| TACGATTGCG | 84 | translation initiation factor eIF-5A | CND05400 | XM_570147 |
| CAGATCTTCT | 81 | guanine nucleotide-binding protein | CNH01270 | XM_572317 |
| GTATCAACGG | 81 | predicted protein | unknown |  |
| CAGGAGATAT | 78 | small GTP-binding protein | CNB03430 | XM_568917 |
| TGGATTGGGG | 78 | NADH-ubiquinone oxidoreductase subunit | CNE03960 | XM_571023 |
| AGATGAATGG | 75 | cytochrome c oxidase subunit IV | CNL04890 | XM_568031 |
| ACTATCAAAA | 75 | no hit |  |  |
| ACCGAGTGAG | 75 | ribosomal protein S19-1 | CNC06100 | XM_569872 |
| TTCGGCAAGG | 74 | ADP, ATP carrier protein | CNM01080 | XM_568302 |
| CCAATGACGT | 73 | GTP-binding nuclear protein Spi1p | CNE02890 | XM_571000 |
| CAACTTGTTA | 72 | glutathione peroxidase HYR1 | CNN00220 | XM_568531 |
| Supplemental Table S3. cont. |  |  |  |  |
| AAGGGTGGTG | 72 | ribosomal protein L24 | CNG02920 | XM_571980 |
| TATATGTGTA | 71 | heat shock protein 12 | CNG04220 | XM_572088 |
| GGCCGACCTG | 71 | ribosomal protein L1 | CNJ03370 | XM_567416 |
| GCCGTTCTTA | 71 | predicted protein | unknown |  |
| ATGGAGTGCA | 71 | ribosomal protein L19 | CNI01090 | XM_572717 |
| TTGTATGATA | 71 | H/ACA ribonucleoprotein complex  subunit 3 | CND01790 | XM_570256 |
| CTTGTAGAAA | 68 | no hit |  |  |
| CTTGTAGCGT | 68 | no hit |  |  |
| CTTCCTGTTC | 67 | phosphoglucomutase | CNN00430 |  |
| TGGATGTGGA | 66 | suppressor protein of mitochondrial  histone mutant | CNM01030 | XM_568570 |
| GATTCATATC | 66 | ribosomal protein S24 | CND04480 | XM_568307 |
| TATATATGCA | 65 | heat shock protein 70 | CNC02320 | XM_570598 |
| TGTTGTTCAA | 63 | expressed protein | CNA02660 | XM_569509 |
| TAATAACAAA | 62 | short-chain dehydrogenase/reductase SDR | CND02230 | XM_566659 |
| GATATTGACT | 59 | multiple genomic contig hits |  |  |
| TAAAGCCGTA | 59 | 2-keto-3-deoxygluconate oxidoreductase | CNA01160 | XM_566558 |
| TAACGCATAA | 58 | hypothetical protein | CNM00840 | XM_568420 |
|  |  |  |  |  |
| Tag | *pka1* (49224) | Predicted product from BLASTx | JEC21 gene ID | NCBI Accession ID |
| GCATTCTTTA | 488 | ribosomal protein s11 | CNA06500 | XM_566991 |
| AGGATGAGAA | 473 | hypothetical protein | CNC00760 | XM_569376 |
| CATACAGGTC | 312 | glutamine synthetase | CNA04370 | XM_566794 |
| TAGTGTCCCG | 290 | 6-phosphogluconate dehydrogenase | CNK01050 | XM_567793 |
| TGTTATCGGT | 284 | heat shock protein 90 homolog | CNM01520 | XM_568451 |
| AAAAAAAAAA* | 268 | multiple hits |  |  |
| GTCGTAGAGT | 260 | enolase | CNC00160 | XM_569379 |
| CATTTCTTCA | 248 | predicted protein | unknown |  |
| TCAGAAGTTG | 243 | thioredoxin | CNC04200 | XM_569667 |
| AGAGATTTCT | 241 | translation elongation factor 2 (TEF2) | CND06220 | XM_570549 |
| AGTACTCTTC | 239 | ribosomal protein P2 | CNF02150 | XM_571539 |
| TGACTGTTTA | 239 | heat shock protein 12 | CND05600 | XM_570476 |
| TATGATAGTG | 221 | ribosomal protein L7a (L8) | CNH02040 | XM_572378 |
| GCAGATCTAT | 214 | ribosomal protein RPL39 | CND05700 | XM_570480 |
| TATACCTATG | 202 | ribosomal protein s3ae (S1) | CNB04880 | XM_568980 |
| CGACAGACCG | 202 | translation elongation factor 1 (TEF1) | CNM01300 | XM_568462 |
| CATTCCTTCA | 187 | hypothetical protein | CAN02520 | XM_566638 |
| ACTACGTTCT | 179 | acidic ribosomal protein p1 | CAN06350 | XM_566980 |
| GGTATTCTAT | 171 | ubiquinol-cytochrome-c reductase subunit IX | CNH02740 | XM_572447 |
| TCTTTGATGT | 163 | ADP, ATP carrier protein | CNM01080 | XM_568302 |
| TCGTCTGAAG | 155 | ribosomal protein L5B | CNC05360 | XM_569733 |
| GTCTCTTTAC | 153 | ribosomal protein L10A | CNE03920 | XM_571019 |
| CACATTCTTT | 153 | ubiquitin/S27a fusion protein [imported] | CND03060 | XM_570192 |
| CATTGAGGTT | 153 | ribosomal protein S22 | CNK02900 | XM_567885 |
| ACATCGATCT | 149 | ribosomal protein L31 | CAN06820 | XM_566913 |
| CATCTGTTGA | 146 | homocitrate synthase | CND01200 | XM_570408 |
| CTTTGGATCA | 143 | ribosomal protein L30/L30A | CAN02210 | XM_567145 |
| Supplemental Table S3. cont. |  |  |  |  |
| TGATGGAAGC | 140 | p21 protein, wos2 | CNK01080 | XM_567796 |
| TACGATTGCG | 135 | translation initiation factor eIF-5A | CND05400 | XM_570147 |
| ATTGAGATGG | 133 | no hit |  |  |
| GAATAGTGGG | 133 | hypothetical protein | CAN07830 | XM_567130 |
| CACTTGGATC | 132 | ribosomal protein L23 | CNK03100 | XM_567903 |
| CCAATGACGT | 131 | GTP-binding nuclear protein Spi1p | CNE02890 | XM_571000 |
| TAACCCAAAT | 130 | ribosomal protein L7 | CAN06360 | XM_566981 |
| CATAATTGGC | 128 | putative heat shock protein | CNM02070 | XM_568283 |
| CACATTGATA | 126 | ribosomal protein L13A | CNM02320 | XM_568413 |
| GGTTTGTACA | 123 | ribosomal protein S29 | CNC04290 | XM_570001 |
| GTTTTATGGA | 121 | heat shock protein 60 | CNB03790 | XM_569211 |
| TCTCTTCCGT | 121 | carboxypeptidase | CND00600 | XM_570309 |
| CATTTATGAA | 120 | 1,4-benzoquinone reductase | CNB04240 | XM_569247 |
| AACCTTGCAT | 120 | ribosomal protein L20A | CNJ01560 | XM_567335 |
| GATATGGATA | 120 | chaperone activator | CAN02890 | XM_566701 |
| GTGTAAATGA | 118 | DNA repair protein rhp54 | CNC03920 | XM_569997 |
| CAGATCTTCT | 114 | guanine nucleotide-binding protein | CNH01270 | XM_572317 |
| ACACGTCTGG | 112 | ATP synthase G chain, mitochondrial | CNC03840 | XM_569622 |
| CATCTTTCCT | 112 | ribosomal protein P0 | CNG00300 | XM_571764 |
| GATGCTTTTT | 112 | ribosomal protein L19B | CNI01090 | XM_572717 |
| TGGATGTGGA | 111 | tricarboxylate carrier | CNM01030 | XM_568307 |
| TATATGTGTA | 109 | heat shock protein 12 | CNG04220 | XM_572088 |
| ACTATCGCCT | 108 | ubiquitin conjugating enzyme UBC1 | CNF02760 | XM_571364 |
| TATTCATAAC | 107 | ribosomal protein L37a | CNC00700 | XM_569366 |
| CCATTGTCAC | 105 | glycine hydroxymethyltransferase-like protein | CNJ00410 | XM_567297 |
| TATTACAGCT | 102 | ribosomal protein L6 | CNE03060 | XM_571207 |
| TATATATGCA | 102 | heat shock protein 70 | CNC02320 | XM_569509 |
| CAAAAACATC | 102 | cytochrome c1 precursor | CNF00630 | XM_571714 |
| TTCGGCAAGG | 101 | ADP, ATP carrier protein | CNM01080 | XM_568302 |
| CACCACATTC | 96 | ribosomal protein L18 | CND03460 | XM_570672 |
| TATGATTTTA | 94 | ribosomal protein S28 | CNG04360 | XM_572112 |
| CAACTTGTTA | 94 | glutathione peroxidase HYR1 | CNN00220 | XM_568531 |
| AGAACTCAAA | 94 | ribosomal protein S6B | CND02780 | XM_570222 |
| AAGTGTGGTA | 91 | transaldolase | CNK03170 | XM_567910 |
| TTATCGATAT | 91 | no hit |  |  |
| GAAAAAAAAA | 91 | no hit |  |  |
| TAGTCTTTCG | 91 | aldehyde reductase (NADPH) | CNB04680 | XM_568958 |
| GCATTGGCGT | 90 | hypothetical protein | CNC01620 | XM_569506 |
| AGATGAATGG | 90 | cytochrome c oxidase subunit IV | CNL04890 | XM_568031 |
| CATTGTGACT | 89 | ribosomal protein S7 | CNI01120 | XM_572711 |
| TGGATGAGAT | 88 | ribosomal protein S3 | CAN01060 | XM_566546 |
| CAGAGATGTG | 86 | nonhistone protein 6 | CNF04730 | XM_571615 |
| AGCATATAAA | 86 | ribosomal protein L9 | CAN00250 | XM_566460 |
| GCTTCACAGA | 86 | ribosomal protein L12 | CNC06990 | XM_569829 |
| ACTATCACAA | 84 | ribosomal protein L27A | CAN07570 | XM_567107 |
| Supplemental Table S3. cont. |  |  |  |  |
| CATCGTTACT | 83 | phosphatidylglycerol/phosphatidylinositol  transfer protein | CND01990 | XM_570234 |
| TGATTGGACT | 83 | asparagine synthase (glutamine-hydrolyzing) | CNH00920 | XM_572287 |
| AAAACAAACT | 81 | cytochrome-c oxidase | CNF01340 | XM_571555 |
| AATGGATTAA | 80 | senescence-associated protein | CNJ00800 | XM_567460 |
| AAGGGATCAT | 80 | hypothetical protein | CAN02750 | XM_566675 |
| AAATGGTTTG | 79 | no hit |  |  |
| CATTTTATGT | 79 | heat shock protein 90 | CNM01520 | XM_568451 |
| TTACACAGCA | 78 | ribosomal protein S19 | CNC06100 | XM_569872 |
| ATCCCGTGGC | 78 | ribosomal protein S14 | CNF00670 | XM_571241 |
| TAATAACAAA | 78 | short-chain dehydrogenase/reductase SDR | CND02230 | XM_570430 |
| ACATTTCCGA | 78 | ribosomal protein L14B | CNJ02260 | XM_567521 |
| CATCACCGGT | 78 | adenylosuccinate synthetase | CNC04750 | XM_569734 |
| TCAGAAGGCA | 78 | no hit |  |  |
| TGGATGGGCA | 77 | ubiquinol-cytochrome c oxidoreductase  subunit VIII | CNC02900 | XM_569987 |
| CAGCAGTCAT | 77 | dihydrolipoamide succinyltransferase | CNB00990 | XM_569105 |
| ACATTGGGTT | 77 | dihydrolipoamide acetyltransferase | CND02450 | XM_570444 |
| CTCTCTATGT | 76 | ribosomal protein S24 | CND04480 | XM_570598 |
| AACGGTTATG | 75 | ribosomal protein L3 | CNK02240 | XM_567698 |
| CATCCCAAAG | 74 | UTP-glucose-1-phosphate uridylyltransferase | CNC03700 | XM_569599 |
| AAAGAAGCCC | 74 | no hit |  |  |
| CTTCCTGTTC | 71 | phosphoglucomutase | CNN00430 | XM_568570 |
| GCTTCCACGA | 71 | ribosomal protein L23A | CNC00350 | XM_569415 |
| AAAGAATTTG | 71 | acetohydroxyacid reductoisomerase | CNF02490 | XM_571347 |
| CATATCACTG | 71 | OV-16 antigen precursor  (*Onchocerca volvulus*) | CNK03430 | XM_567927 |
| GATGCTCTGT | 70 | ribosomal protein RPL39 | CND05700 | XM_570480 |
| TAGGCCGTCT | 70 | heat shock protein 10 | CNB03800 | XM_569027 |
| ATAAAAAAAA | 68 | no hit |  |  |
| TATACATTCA | 68 | hypothetical protein | CNB03460 | XM_568923 |

* Some of the AAAAAAAAAA tags are likely to be an artifact of the SAGE library preparation procedure.
