# Supplementary material for: Transcriptional Regulation by Protein Kinase A in Cryptococcus neoformans
Source: PLoS Pathog. 2007 Mar 16;3(3):e42. doi: 10.1371/journal.ppat.0030042 (PMC1828699; doi:10.1371/journal.ppat.0030042)
Supplement: Supplementary file 4 [file ppat.0030042.st004.doc]

| Supplemental Table S4. Tags for ribosome biogenesis genes and related functions | | | | | | | | | |
| --- | --- | --- | --- | --- | --- | --- | --- | --- | --- |
|  |  |  |  |  |  |  |  |  | |
| Tag | WT (49224) | *pkr1* (49224) | *pka1* (49224) | p-value *pkr1* vs WT | p-value  *pka1* vs WT | p-value *pkr1* vs *pka1* | Predicted product based on BLASTx | JEC21 gene ID | NCBI Accession ID |
| GATGCTTTTT | 160 | 133 | 112 | 9.00E-02 | **5.91E-04** | 2.24E-01 | ribosomal protein L19 | CNI01090 | XM_572717 |
| CATTGTGACT | 157 | 135 | 89 | 1.63E-01 | **2.39E-07** | **3.86E-04** | ribosomal protein S7 | CNI01120 | XM_572711 |
| AGCATATAAA | 125 | 46 | 86 | 6.06E-11 | **1.74E-03** | **1.27E-04** | ribosomal protein L9 | CNA00250 | XM_566460 |
| GGCCGACCTG | 121 | 71 | 53 | **1.16E-04** | **9.23E-10** | 7.54E-02 | ribosomal protein L1 | CNJ03370 | XM_567416 |
| GGCTTTCGAA | 60 | 57 | 35 | 7.60E-01 | **1.69E-03** | **4.18E-03** | structural constituent of ribosome | CNB02420 | XM_569167 |
| CAGAACCCCG | 26 | 20 | 12 | 3.35E-01 | **4.23E-03** | 9.69E-02 | ribosomal protein S18 | CNJ03110 | XM_567390 |
| ACCAAGCTTG | 19 | 9 | 5 | 4.10E-02 | **4.82E-04** | 2.43E-01 | ribosomal protein S8 | CNG03720 | XM_572078 |
| AAGCCCGTTG | 17 | 9 | 5 | 9.20E-02 | **1.92E-03** | 2.43E-01 | ribosomal protein s10 | CNF01650 | XM_571499 |
| CTGAAAAAAA | 11 | 4 | 3 | 6.14E-02 | **9.32E-03** | 6.14E-01 | translation initiation factor 3 | CNA03090 | XM_566736 |
| GGCCCATTGT | 8 | 2 | 0 | 6.86E-02 | **6.16E-04** | 1.47E-01 | transcription initiation factor | CNG00450 | XM_571746 |
| ACTACGTTCT | 283 | 603 | 179 | **7.13E-33** | **6.24E-09** | **7.04E-91** | acidic ribosomal protein p1 | CNA06350 | XM_566980 |
| AGTACTCTTC | 215 | 525 | 239 | **2.59E-37** | 1.73E-01 | **3.15E-35** | ribosomal protein P2 | CNF02150 | XM_571539 |
| GCATTCTTTA | 368 | 523 | 488 | **1.18E-08** | 1.06E-06 | 1.09E-01 | ribosomal protein s11 | CNA06500 | XM_566991 |
| ACATCGATCT | 281 | 403 | 149 | **6.80E-08** | **2.59E-13** | **4.85E-38** | ribosomal protein L31 | CNA06820 | XM_566913 |
| GCAGATCTAT | 189 | 384 | 214 | **1.61E-19** | 1.41E-01 | **3.52E-18** | ribosomal protein L39p | CND05700 | XM_570480 |
| TATGATAGTG | 160 | 346 | 221 | **3.53E-20** | **1.91E-04** | **3.28E-10** | ribosomal protein L7a (L8) | CNH02040 | XM_572378 |
| TATACCTATG | 134 | 303 | 202 | **2.50E-19** | **9.75E-06** | **1.86E-07** | ribosomal protein s3ae (S1) | CNB04880 | XM_568980 |
| CACTTGGATC | 201 | 258 | 132 | **3.48E-03** | **6.41E-06** | **1.46E-13** | ribosomal protein L23 | CNK03100 | XM_567903 |
| AACCTTGCAT | 116 | 253 | 120 | **2.50E-15** | 8.04E-01 | **7.20E-15** | ribosomal protein L20A | CNJ01560 | XM_567335 |
| CACCACATTC | 106 | 251 | 96 | **9.86E-18** | 3.95E-01 | **5.69E-22** | ribosomal protein L18 | CND03460 | XM_570672 |
| CTTTGGATCA | 204 | 243 | 143 | 4.14E-02 | **1.16E-04** | **3.00E-09** | ribosomal protein L30/L30A | CNA02210 | XM_567145 |
| GGTTTGTACA | 108 | 225 | 123 | **1.08E-12** | 2.37E-01 | **4.44E-10** | ribosomal protein S29-A (S36) | CNC04290 | XM_570001 |
| TTACACAGCA | 78 | 225 | 78 | **1.19E-21** | 9.29E-01 | **1.13E-21** | putative ribosomal protein S19 | CNC06100 | XM_569872 |
| TAACCCAAAT | 149 | 208 | 130 | **6.00E-04** | 1.70E-01 | **8.41E-08** | ribosomal protein L7 | CNA06360 | XM_566981 |
| ACATTTCCGA | 202 | 202 | 78 | 9.72E-01 | **3.94E-19** | **1.96E-18** | ribosomal protein L14B | CNJ02260 | XM_567521 |
| GTCTCTTTAC | 100 | 186 | 153 | **1.63E-08** | **5.69E-05** | 5.66E-02 | ribosomal protein L10A | CNE03920 | XM_571019 |
| TATTCATAAC | 89 | 184 | 107 | **1.41E-10** | 1.13E-01 | **2.33E-07** | ribosomal protein L37a | CNC00700 | XM_569366 |
| TCGTCTGAAG | 102 | 161 | 155 | **5.83E-05** | **7.83E-05** | 7.81E-01 | ribosomal protein L5B | CNC05360 | XM_569733 |
| GATGCTCTGT | 60 | 161 | 70 | **1.64E-14** | 2.82E-01 | **4.48E-12** | ribosomal protein RPL39 | CND05700 | XM_570480 |
| GCTTCACAGA | 119 | 155 | 86 | 1.75E-02 | 6.00E-61 | **3.81E-07** | ribosomal protein L12 | CNC06990 | XM_569829 |
| ACTATCACAA | 62 | 147 | 84 | **4.01E-11** | 3.06E-02 | **3.27E-06** | ribosomal protein L27A | CNA07570 | XM_567107 |
| CACATTGATA | 52 | 140 | 126 | **9.24E-13** | **2.04E-11** | 5.09E-01 | ribosomal protein L13A | CNM02320 | XM_568413 |
| CATCTTTCCT | 108 | 137 | 112 | 4.24E-02 | 7.30E-01 | 5.68E-02 | ribosomal protein P0 | CNG00300 | XM_571764 |
| CTCTCTATGT | 86 | 136 | 76 | **2.11E-04** | 3.74E-01 | **1.09E-06** | ribosomal protein S24 | CND04480 | XM_570598 |
| AGAACTCAAA | 94 | 124 | 94 | 2.67E-02 | 9.61E-01 | **6.77E-03** | ribosomal protein S6B | CND02780 | XM_570222 |
| ATCCCGTGGC | 121 | 123 | 78 | 8.90E-01 | **3.20E-04** | **2.69E-03** | ribosomal protein S14 | CNF00670 | XM_571241 |
| TAAATGGTGC | 72 | 120 | 50 | **1.34E-04** | 2.04E-02 | **2.95E-10** | ribosomal protein RPL22 | CND05910 | XM_570540 |
| CTATTGGTCG | 108 | 113 | 30 | 7.07E-01 | **3.17E-16** | **1.85E-17** | ribosomal protein S8 | CNG03720 | XM_572078 |
| AAGCGATTTT | 96 | 111 | 58 | 2.37E-01 | **3.49E-04** | **1.23E-05** | ribosomal protein S26 | CNH01770 | XM_572351 |
| GCTTCCACGA | 71 | 107 | 71 | **2.68E-03** | 9.96E-01 | **2.08E-03** | ribosomal protein L23A | CNC00350 | XM_569415 |
| ACCGAGTGAG | 26 | 75 | 29 | **5.15E-08** | 6.90E-01 | **2.06E-07** | ribosomal protein S19-1 | CNC06100 | XM_569872 |
| AAGGGTGGTG | 71 | 72 | 46 | 9.38E-01 | **5.62E-03** | 1.13E-02 | ribosomal protein L24 (L30) | CNG02920 | XM_571980 |
| ATGGAGTGCA | 44 | 71 | 31 | **6.13E-03** | 6.83E-02 | **1.19E-05** | ribosomal protein L19 | CNI01090 | XM_572717 |
| TTGTATGATA | 21 | 71 | 30 | **5.22E-09** | 1.81E-01 | **4.71E-06** | H/ACA ribonucleoprotein complex subunit 3 (snoRNP protein NOP10) | CND01790 | XM_570256 |
| TCTGTCGAGG | 24 | 33 | 9 | 1.99E-01 | 1.15E-03 | **2.12E-05** | ribosomal protein S12 | CNC03760 | XM_569612 |
| AAAGAGTCTG | 12 | 30 | 13 | **2.64E-03** | 9.05E-01 | **4.10E-03** | ribosomal protein S4-1 | CNA06200 | XM_567206 |
| AAAGCGATGC | 12 | 29 | 7 | **2.55E-03** | 2.22E-01 | **4.36E-05** | ribosomal protein S9 | CNE02160 | XM_570885 |
| GGCTTCGGTC | 1 | 19 | 1 | **4.44E-07** | 5.86E-01 | **4.58E-06** | acidic ribosomal protein p1-alpha | CNF02150 | XM_571539 |
| CACAAGCCCC | 14 | 17 | 4 | 5.17E-01 | 8.00E-03 | **2.07E-03** | ribosomal protein L22 | CNE04980 | XM_571022 |
| AGGACGGATG | 0 | 14 | 4 | **3.02E-06** | 1.36E-02 | **3.76E-03** | ribosomal protein L34-like protein | CNA07980 | XM_567178 |
| ATATACATCT | 3 | 11 | 9 | 1.22E-02 | 4.05E-02 | 5.44E-01 | ribosomal protein 16 | CNB02750 | XM_568864 |
| TAAAAATGTT | 5 | 5 | 0 | 9.06E-01 | **9.12E-03** | 1.08E-02 | S-phase specific ribosomal protein cyc07 | CNB04880 | XM_568980 |

Statistically significant p-values are indicated in bold.
