# Supplementary material for: Transcriptional Regulation by Protein Kinase A in Cryptococcus neoformans
Source: PLoS Pathog. 2007 Mar 16;3(3):e42. doi: 10.1371/journal.ppat.0030042 (PMC1828699; doi:10.1371/journal.ppat.0030042)
Supplement: Supplementary file 5 [file ppat.0030042.st005.doc]

| **Supplemental Table S5. Tags for genes related to carbohydrate and amino acid metabolism, cytoskeleton and vacuolar function.** | | | | | | | | | |
| --- | --- | --- | --- | --- | --- | --- | --- | --- | --- |
|  |  |  |  |  |  |  |  |  | |
| Tag | WT (49224) | *pkr1* (49,224) | *pka1* (49224) | p-value *pkr1* vs WT | p-value *pka1* vs WT | p-value  *pka1* vs *pkr1* | Predicted product from BLASTx | JEC21 gene ID | NCBI Accession ID |
|  | |  |  |  |  |  |  |  |  |
| Carbohydrate metabolism | |  |  |  |  |  |  |  |  |
| CACATCGTGC | 105 | 33 | 66 | **2.12E-11** | **3.88E-04** | **2.85E-04** | mitochondrial isocitrate dehydrogenase1 | CNJ02060 | XM_567378 |
| TACTTGACCA | 68 | 13 | 18 | **3.46E-12** | **8.74E-12** | 3.60E-01 | UDP-N-acetylglucosamine diphosphorylase | CNF01520 | XM_571302 |
| GACATATGAA | 62 | 54 | 6 | 4.55E-01 | **5.30E-18** | **8.57E-14** | alpha-amylase AmyA | CNH03660 | XM_572528 |
| TGTTATATGA | 25 | 8 | 14 | **1.66E-03** | 4.73E-02 | 1.66E-01 | inositol-1(or 4)-monophosphatase, putative | CNG03150 | XM_572023 |
|  |  |  |  |  |  |  |  |  |  |
| ATAAGCTTTC | 30 | 120 | 38 | **6.52E-17** | 2.70E-01 | **7.62E-14** | mannitol-1-phosphate dehydrogenase | CNG00600 | XM_571772 |
| TAAAGCCGTA | 28 | 59 | 24 | **2.73E-04** | 4.51E-01 | **1.14E-05** | 2-keto-3-deoxygluconate oxidoreductase | CNA01160 | XM_566558 |
|  |  |  |  |  |  |  |  |  |  |
| TAGTGTCCCG | 173 | 114 | 290 | **1.03E-04** | **1.83E-10** | **2.63E-21** | 6-phosphogluconate dehydrogenase | CNK01050 | XM_567793 |
| GTCGTAGAGT | 192 | 173 | 260 | 2.90E-01 | **1.34E-04** | **1.45E-07** | phosphopyruvate hydratase, enolase 3 | CNC00160 | XM_569379 |
| CATCTGTTGA | 22 | 34 | 146 | 8.03E-02 | **2.57E-33** | **5.73E-20** | homocitrate synthase | CND01200 | XM_570408 |
| ACATTGGGTT | 40 | 36 | 77 | 6.24E-01 | **4.51E-05** | **9.37E-06** | dihydrolipoyllysine-residue acetyltransferase | CND02450 | XM_570444 |
| CAGCAGTCAT | 17 | 51 | 77 | **4.36E-06** | **3.29E-14** | **5.88E-03** | dihydrolipoamide succinyltransferase | CNB00990 | XM_569105 |
| CATCCCAAAG | 40 | 51 | 74 | 2.09E-01 | **1.42E-04** | 1.60E-02 | UTP-glucose-1-phosphate uridylyltransferase | CNC03700 | XM_569599 |
| AAAAACGCGT | 55 | 19 | 64 | **5.09E-06** | 3.45E-01 | **3.12E-08** | myo-inositol 1-phosphate synthase | CNC06440 | XM_569900 |
| CAGCCAGTGT | 15 | 21 | 56 | 2.84E-01 | **3.22E-09** | **1.29E-05** | aldehyde dehydrogenase | CNE04360 | XM_571099 |
| AACTTTGTTC | 33 | 10 | 53 | **1.44E-04** | 1.02E-02 | **4.97E-10** | isocitrate dehydrogenase | CNA04610 | XM_566837 |
| ACACTTGAAT | 24 | 14 | 51 | 8.22E-02 | **2.06E-04** | **4.69E-07** | saccharopine dehydrogenase (NAD+, L-lysine-forming) | CND06290 | XM_570078 |
| AGCATTTTAA | 3 | 14 | 48 | **1.71E-03** | **1.31E-16** | **1.42E-06** | succinate dehydrogenase flavoprotein subunit precursor | CNI03270 | XM_572631 |
| TTGGCTCCCA | 13 | 4 | 45 | 2.14E-02 | **2.98E-07** | **4.57E-11** | fumarase | CNC01700 | XM_569517 |
| TTCCCTAATC | 21 | 11 | 44 | 5.40E-02 | **8.91E-04** | **4.98E-07** | fructose-bisphosphatase | CNA00470 | XM_566475 |
| CAGCAGTGTA | 6 | 16 | 37 | 2.05E-02 | **4.37E-09** | **7.51E-04** | indole-3-acetaldehyde dehydrogenase | CNF03900 | XM_571348 |
| ATGCATATGA | 17 | 26 | 33 | 1.11E-01 | **5.22E-03** | 3.35E-01 | UDP-galactose 4-epimerase | CNA08350 | XM_567074 |
| CAAAAGTAGA | 11 | 11 | 32 | 9.78E-01 | **9.54E-05** | **3.84E-04** | pyruvate carboxylase | CNF00650 | XM_571482 |
| ACTCAGGTTG | 2 | 3 | 32 | 6.16E-01 | **5.72E-11** | **4.31E-08** | fructose-bisphosphate aldolase | CNB00300 | XM_568771 |
| CAAACATCTA | 12 | 12 | 29 | 9.25E-01 | **1.35E-03** | **4.01E-03** | D-lactate dehydrogenase | CNK01540 | XM_567828 |
| TACAATGAAA | 6 | 0 | 28 | 1.75E-02 | **1.74E-06** | **2.55E-10** | pyruvate dehydrogenase (acetyl-transferring) | CNA03850 | XM_566723 |
| CCAATGATTA | 1 | 12 | 32 | 7.30E-04 | **3.51E-12** | **5.92E-04** | aconitate hydratase | CND02620 | XM_570245 |
| GAAGTAGAAA | 12 | 6 | 30 | 1.23E-01 | **1.11E-03** | **5.89E-06** | L-malate dehydrogenase | CNG03100 | XM_572013 |
| AAGCTATGAA | 10 | 22 | 27 | 1.48E-02 | **7.28E-04** | 4.32E-01 | phosphoglyceromutase | CNK00280 | XM_567598 |
| TCTTTCCGAG | 8 | 6 | 24 | 6.89E-01 | **5.14E-04** | **5.22E-04** | glyceraldehyde-3-phosphate dehydrogenase | CNF03160 | XM_570436 |
| CATTTTCTCA | 0 | 7 | 21 | **1.57E-03** | **1.03E-09** | **5.63E-03** | succinate dehydrogenase iron-sulfur subunit | CNG03480 | XM_572036 |
| TTATTTCATA | 3 | 2 | 12 | 7.98E-01 | **3.26E-03** | **4.43E-03** | ribose-5-phosphate isomerase | CND05060 | XM_567487 |
| GATCGGATAC | 2 | 2 | 12 | 9.50E-01 | **1.94E-03** | **6.91E-03** | 6-phosphofructokinase | CNJ01080 | XM_568853 |
| TGTGAATGTG | 3 | 3 | 11 | 8.55E-01 | **9.23E-03** | 3.11E-02 | hexokinase | CNB02660 | XM_569379 |
| TACGCTATAC | 1 | 1 | 7 | 7.31E-01 | **5.20E-03** | 2.87E-02 | succinate dehydrogenase flavoprotein subunit precursor | CNI03270 | XM_572631 |
| **Amino acid metabolism** | |  |  |  |  |  |  |  |  |
| GAACTGGCGG | 36 | 27 | 9 | 2.32E-01 | **6.19E-07** | **3.52E-04** | adenosylhomocysteinase | CND00240 | XM_570337 |
| TGTAGGGCTG | 30 | 25 | 14 | 5.06E-01 | **5.98E-03** | 6.38E-02 | methionine adenosyltransferase | CNA04060 | XM_566752 |
| CATTGGGTAT | 12 | 8 | 4 | 3.19E-01 | **8.74E-03** | 1.55E-01 | aminomethyltransferase | CNC04350 | XM_569702 |
| GATGCACCTG | 78 | 102 | 12 | 6.83E-02 | **2.50E-11** | **2.09E-15** | guanosine-diphosphatase | CNB04810 | XM_568971 |
| TAGCTCTTTT | 15 | 34 | 6 | 4.86E-01 | **3.08E-03** | **1.13E-03** | adenine phosphoribosyltransferase | CND05020 | XM_570064 |
| CCATCATCAT | 4 | 15 | 6 | 5.10E-03 | 4.08E-01 | 2.66E-02 | aminomethyltransferase | CNA03770 | XM_566711 |
| CCATTGTCAC | 30 | 44 | 105 | 8.32E-02 | **4.63E-15** | **9.54E-09** | glycine hydroxymethyltransferase | CNJ00410 | XM_567297 |
| TGATTGGACT | 27 | 30 | 83 | 6.48E-01 | **9.59E-11** | **1.71E-08** | asparagine synthase (glutamine-hydrolyzing) | CNH00920 | XM_572287 |
| CATCACCGGT | 40 | 31 | 78 | 2.52E-01 | **3.36E-05** | **8.01E-07** | adenylosuccinate synthase | CNC04750 | XM_569734 |
| AAAGAATTTG | 35 | 47 | 71 | 1.30E-01 | **2.65E-05** | 1.40E-02 | acetohydroxyacid reductoisomerase | CNF02490 | XM_571347 |
| TATTCCGGTC | 23 | 30 | 65 | 2.74E-01 | **5.75E-08** | **4.48E-05** | argininosuccinate synthase | CND00700 | XM_570386 |
| TAGATTCTTT | 30 | 18 | 53 | 7.22E-02 | **2.07E-03** | **3.65E-06** | phenylalanine-tRNA ligase | CNJ02270 | XM_567522 |
| TACAAATCAA | 14 | 11 | 39 | 5.72E-01 | **3.18E-05** | **1.06E-05** | pyrimidine synthesis protein | CNA06000 | XM_566954 |
| GGCCGCGCTA | 10 | 10 | 39 | 9.03E-01 | **3.04E-07** | **2.23E-05** | asparagine-tRNA ligase | CNE01390 | XM_570734 |
| GTCTCTCACT | 10 | 9 | 33 | 9.04E-01 | **1.39E-05** | **6.76E-05** | arginine-specific carbamoyl-phosphate synthetase,ammonia chain | CNG02810 | XM_571945 |
| CAAAACGAAA | 13 | 23 | 27 | 6.91E-02 | **7.77E-03** | 5.64E-01 | glycine dehydrogenase (decarboxylating) | CNN00460 | XM_568576 |
| GTTGAGAAGC | 6 | 7 | 23 | 6.21E-01 | **6.77E-05** | **9.29E-04** | chimeric spermidine synthase/saccharopine dehydrogenase | CNG01150 | XM_571786 |
| TATACACAGA | 8 | 10 | 22 | 6.38E-01 | **3.63E-03** | 3.09E-02 | cysteine synthase | CNL05880 | XM_568121 |
| GCACTTAATT | 5 | 15 | 21 | 1.94E-02 | **1.20E-04** | 3.13E-01 | phospho-2-dehydro-3-deoxyheptonate aldolase | CNL06640 | XM_568187 |
| Cytoskeleton |  |  |  |  |  |  |  |  |  |
| CAGTACCAGG | 2 | 3 | 17 | 6.16E-01 | **3.24E-05** | **7.30E-04** | β - tubulin | CNC03260 | XM_569650 |
| TCTTGTTTCG | 1 | 1 | 7 | 7.31E-01 | **5.20E-03** | 2.87E-02 | α - tubulin | CNB02810 | XM_568870 |
| Vacuolar function | |  |  |  |  |  |  |  |  |
| ACATTTTGTG | 15 | 13 | 32 | 6.83E-01 | **2.95E-03** | **2.25E-03** | vacuolar ATP synthase | CNI01180 | XM_572836 |
| TATGAACGCT | 1 | 3 | 9 | 3.84E-01 | **5.91E-03** | 9.66E-02 | vacuolar ATP synthase subunit d | CNC00590 | XM_569347 |
| TAATCACACA | 1 | 0 | 7 | **8.08E-04** | **5.20E-03** | 4.48E-01 | vacuole fusion, non-autophagic-related protein | CNA05660 | XM_566888 |
| CATATATTAC | 1 | 1 | 6 | 3.78E-01 | **9.78E-03** | 4.57E-02 | vacuole protein | CNM00250 | XM_568436 |

Statistically significant P-values are indicated in bold.
