# Supplementary material for: Transcriptional Regulation by Protein Kinase A in Cryptococcus neoformans
Source: PLoS Pathog. 2007 Mar 16;3(3):e42. doi: 10.1371/journal.ppat.0030042 (PMC1828699; doi:10.1371/journal.ppat.0030042)
Supplement: Supplementary file 6 [file ppat.0030042.st006.doc]

| Supplemental Table S6. Primers used in real-time PCR analysis | | | |
| --- | --- | --- | --- |
|  |  |  |  |
| JEC21 gene ID | NCBI accession ID | Primer sequence (5'-3') |  |
|  |  |  |  |
| CNA00580 | XM_567231 | AAACCCTCAGCAGACGAATACAA |  |
|  |  | CGCCAAAGCCGATAGCA |  |
| CNC05350 | XM_569731 | AGGAGGGACAAGGCGAGAAG |  |
|  |  | CATCGCTTGAGCCCTAGCA |  |
| CNN00330 | XP_568551 | CCGCGATGTCCCCATTC |  |
|  |  | CATGACCACGACGATACCAATT |  |
| CNB02680 | XM_568855 | TGAAAGCCGGCAAGGTAGA |  |
|  |  | GTTGACCTCGGACTCGAGACA |  |
| CNF03900 | XM_571348 | GCCGCCAACGACTCTGTCTA |  |
|  |  | GCTGGCTCGAGCGATATTG |  |
| CNJ02970 | XP_567576 | TTCCCTCGGATCACCATTTG |  |
|  |  | GCGGAGCTTAGGCCGAAT |  |
| CNC00760 | XM_569376 | GCTGTCGTCGCCGATGTC |  |
|  |  | CGATGGAAAGGTCAAGAGAGTTG |  |
| *OVA1* (CNA03430) | XM_567927 | GCTCGTCAACTCGGCTAGTCTT |  |
|  |  | CGGTCCAGTTGACAGCGTAA |  |
| *ACT1* (CNA04650) | XP_566845 | CCACACTGTCCCCATTTACGA |  |
|  |  | CAGCAAGATCGATACGGAGGAT |  |
| *GPD1* (CNF03160) | XP_571627 | GTCTGCGGTGTCAACCTTGAT |  |
|  |  | CAAGAAGCGTTGGACACGATT |  |
|  |  |  |  |

Note that the primer sequences are for the genes in the serotype A strain H99; the gene identifiers refer to the orthologs of these genes in the JEC21 genome.
